# Conformational transitions induced by γ-amino butyrate binding in GabR, a bacterial transcriptional regulator

**DOI:** 10.1038/s41598-019-55581-1

**Published:** 2019-12-17

**Authors:** Mario Frezzini, Leonardo Guidoni, Stefano Pascarella

**Affiliations:** 1 Department of Biochemical Sciences “A Rossi Fanelli”, Sapienza, University of Rome, 00185 Rome, Italy; 2 Department of Physics and Chemistry Sciences, University of L’Aquila, 67100 L’Aquila, Italy; 3 Department of Information Engineering, Computer Science and Mathematics, University of L’Aquila, 67100 L’Aquila, Italy

**Keywords:** Computational models, Structural biology, Computational biology and bioinformatics, Biochemistry, Transcription factors

## Abstract

GabR from *Bacillus subtilis* is a transcriptional regulator of the MocR subfamily of GntR regulators. The MocR architecture is characterized by the presence of an N-terminal winged-Helix-Turn-Helix domain and a C-terminal domain folded as the pyridoxal 5′-phosphate (PLP) dependent aspartate aminotransferase (AAT). The two domains are linked by a peptide bridge. GabR activates transcription of genes involved in γ-amino butyrate (GABA) degradation upon binding of PLP and GABA. This work is aimed at contributing to the understanding of the molecular mechanism underlying the GabR transcription activation upon GABA binding. To this purpose, the structure of the entire GabR dimer with GABA external aldimine (holo-GABA) has been reconstructed using available crystallographic data. The structure of the apo (without any ligand) and holo (with PLP) GabR forms have been derived from the holo-GABA. An extensive 1 μs comparative molecular dynamics (MD) has been applied to the three forms. Results showed that the presence of GABA external aldimine stiffens the GabR, stabilizes the AAT domain in the closed form and couples the AAT and HTH domains dynamics. Apo and holo GabR appear more flexible especially at the level of the HTH and linker portions and small AAT subdomain.

## Introduction

The molecular architecture of the transcription factors (TF) of the family GntR consists of two domains. The N-terminal one, about 60 residue long, is folded as the winged-Helix-Turn-Helix architecture (wHTH) and functions as a DNA recognition and binding^1,2^ module. The larger C-terminal domain belongs to one of at least four different folds and provides oligomerization and/or effector binding functions. A GntR subfamily was named MocR after designation of the regulator of rhizopine catabolism genes^3^. This subfamily is characterized by a large C-terminal domain (about 350 residues) folded as the type-I pyridoxal 5′-phosphate (PLP)-dependent enzymes^4^. Aspartate aminotransferase (AAT)^5^ is the reference structure for this fold. The fold is structured in two domains, a large N-terminal one containing a seven-stranded β-sheet and a small one comprising the C-terminal part of the peptide chain. These two domains will be referred to as “subdomains” throughout the article to distinguish them from the whole AAT domain. The small subdomain is folded into a three-stranded β-sheet covered with helices on one side. The active site is located in a cleft between the two subdomains^6^. Often, PLP-dependent enzymes of fold type-I are homodimeric. Most of the fold type-I enzymes undergo a transition from an open to a closed conformation upon substrate binding. This change, described in detail for the aspartate aminotransferase^7^, consists in the closure of the small and large subdomains and it is deemed to be essential for catalysis.

The MocR wHTH and AAT domains are linked to each other by a peptide bridge of variable length in different MocRs^8,9^. MocRs are widespread among several bacterial phyla^10^ and may be divided into subfamilies^11–13^ on the basis of AAT domain similarity.

Several MocR regulators have been studied and characterized. For example: TauR from *Rhodobacter capsulatus*^14^*;* GabR from *Bacillus subtilis*; PtsJ from *Salmonella typhimurium*^15^; PdxR from *Corynebacterium glutamicum*^16^, *Streptococcus pneumoniae*^17^, *Listeria monocytogenes*^18^, *Streptococcus mutans*^19^, *Bacillus clausii*^20^. Recently, a new *Brevibacillus brevis* MocR has been demonstrated to regulate the gene coding for d-alanyl-d-alanine ligase^21^. In general, the MocR regulators are involved in the control of the expression of genes connected directly or indirectly to PLP functions. Indeed, TauR activates the expression of taurine utilization genes which include a PLP-dependent taurine-pyruvate aminotransferase^14^. GabR bound to PLP and γ-amino butyric acid (GABA) activates transcription of genes coding for the PLP-dependent GABA aminotransferase and succinic semi-aldehyde dehydrogenase. PtsJ regulates the production of pyridoxal kinase, involved in the PLP salvage pathway^22^. PdxR is involved in the regulation of the PLP synthesis. Other examples are reported in a recent review^23^. However, a few subgroups of MocR regulators^24^ were predicted to regulate genes coding for different types of proteins, such as membrane transporters.

The so-far best characterized MocR regulator is GabR from *Bacillus subtilis*^4,25^. Several experimental studies have demonstrated that GabR is dimeric and can bind to DNA in absence of co-regulators but must interact with PLP and GABA to activate *gabTD* gene transcription. *In vitro* experiments have also shown that it acts directly as an autorepressor of *gabR* gene^26^. Spectroscopic studies^27^ have shown that GABA forms an external aldimine with the PLP in the aminotransferase domain of GabR. PLP reacts with a lysine in the active site of the GabR forming the internal aldimine. The dimeric GabR tertiary structure has been solved in the apo form (with imidazole at the AAT domain active site) and in holo form as an internal aldimine complex with PLP^4,28^. More recently the structure of the AAT-like domain of GabR complexed with PLP-GABA external aldimine (holo-GABA) have been solved independently by two groups^27,29^. A careful comparison of the crystallographic structures of the holo and holo-GABA AAT domains of GabR, suggested that GABA binding triggers a subdomain closure like the conformational change observed in AAT from *E. coli*. This conformational change is considered an important component of the mechanism of transcription activation by GABA binding.

Although structural and functional characterization of GabR and other MocRs is proceeding at a fast pace, not much is known about the molecular mechanisms underlying the effector binding and its influence on the functional properties of the regulator. Several experimental approaches able to tackle this problem are being applied, such as Atomic Force Microscopy^30^. In addition to that, theoretical techniques such as molecular dynamics (MD) can provide insights into the conformational changes induced by effector binding. This approach is now routinely and widely used to simulate and study the conformational changes of proteins and protein complexes as, for example, lobe motions or allosteric transitions^31^. A 200 and 350 ns molecular dynamics simulation on the apo (without PLP), and holo (PLP internal aldimine) forms of the GabR from *Bacillus subtilis* has been recently carried out^32^. The main results of the work suggested that the presence of PLP bound at the active site as an internal aldimine increases the flexibility of the GabR. Apparently, linkers and HTH domains are the most affected portions of the protein. The recent availability of the crystallographic structures of the GabR AAT domains complexed with GABA-external aldimine^27,29^ opened new and interesting possibilities for further MD studies. In consideration of the fact that the solved structure was lacking the HTH domain, we have rebuilt the entire complex using homology modeling. The obtained structure represents the holo-GABA GabR complex. From this model, the structures of other two GabR forms have been derived: the apo (no ligand) and the holo (with PLP internal aldimine) forms. In this work, the apo GabR form represents a reference structure to which compare the properties of the holo and holo-GABA forms. It is not clear whether apo GabR exists *in vivo* and whether it has a significant physiological role within the bacterial cell. This study reports on a 1 μs MD simulation carried out on the three GabR forms, apo, holo and holo-GABA (PLP-GABA external aldimine). A comparison between the molecular dynamics trajectories calculated for the three forms of GabR confirmed that the presence of only PLP at the active site destabilizes the interaction between the wHTH domain of one subunit and the AAT domain of the other. Apparently, the effect of the GABA external aldimine implies a partial stiffening of GabR AAT domains and a significant alteration of their dynamics and interactions with the HTH domains. Possible relevance of these observations to the transition from the open to closed conformation will be considered and discussed.

## Results

### Homology modelling

**Figure 1:**
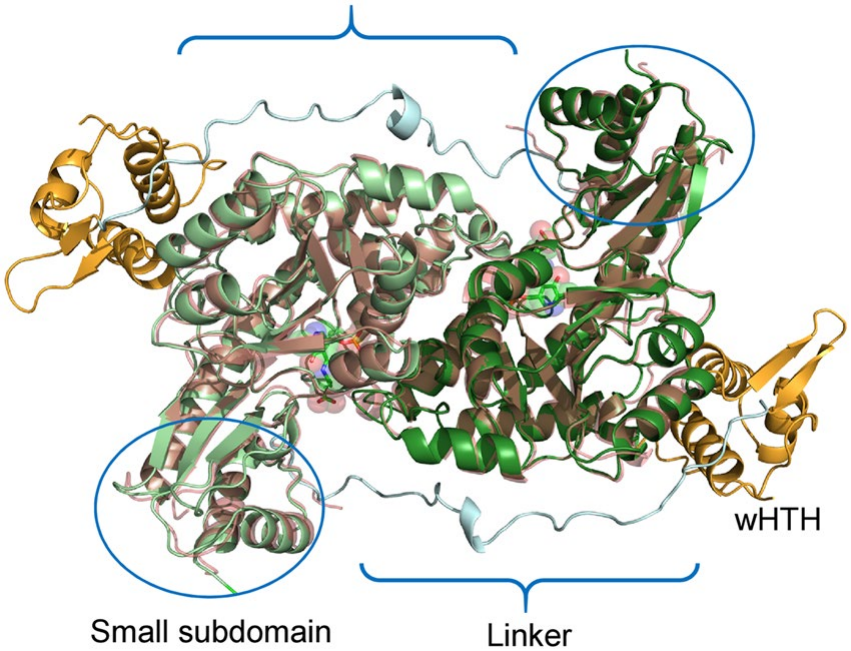
Structure of holo-GABA GabR dimer. Cartoon colors indicate the different domains in the two subunits: orange indicate the wHTH domains; pale cyan the two linkers; dark and light green the AAT domains. PLP-GABA adducts are displayed as stick models enclosed in transparent space filling representation. Ovals enclose the small subdomain of each AAT domain. Curley brackets highlight the linker position. Structure of the 5T4J AAT domains, superposed to the corresponding holo-GABA GabR chains, is displayed as light pink transparent cartoon. This figure has been created with the software open-source PyMOL v. 1.8.4.0 (https://pymol.org/)^55^.

The construction of the holo-GABA ternary complex was carried out through homology modelling implemented in the program Modeller^33^. A chimeric model was built by using as a template the segment 1–104 of 4N0B containing the wHTH and the linker domains, and the AAT domain from 5T4J covering positions 105–469 of the final model (Supplementary Fig. S1). An unresolved segment in 5T4J encompassed by positions 433–437 of GabR was reconstructed during homology modelling taking the coordinates from the corresponding region available in 4N0B. Likewise, side chain conformation of arginine residues in positions 138 and 271 missing in 5T4J, were taken from the template. The dimeric form of GabR has been modelled on the quaternary asset of 4N0B. Quality checks of the model relied on QMEAN^34^ server using the QMEANDisCo^35^ method. The results validated the holo-GABA GabR model (Supplementary Fig. S2): the global score obtained for the holo-GABA model was 0.85 ± 0.05, close to that obtained for the template 5T4J which was equal to 0.88 ± 0.05. Reconstructed holo-GABA ternary complex is shown in Fig. 1 compared with the AAT domains of the template 5T4J. The apo and holo forms have been derived from this structure as reported in Materials and Methods section to assure homogeneity of MD simulations and result comparability (Table 1).

**Table 1:** GabR forms used in MD.

Denomination	characteristics
holo-GABA	GabR in complex with PLP-GABA external aldimine. wHTH and linker modules from structure 4N0B and AAT domains from 5T4J. Closed conformation.
holo	Derived from holo-GABA after GABA removal. PLP internal aldimine reconstructed according to its structure in 4N0B.
apo	Holo-GABA after PLP-GABA complex removal.

### Molecular dynamics

**Figure 2:**
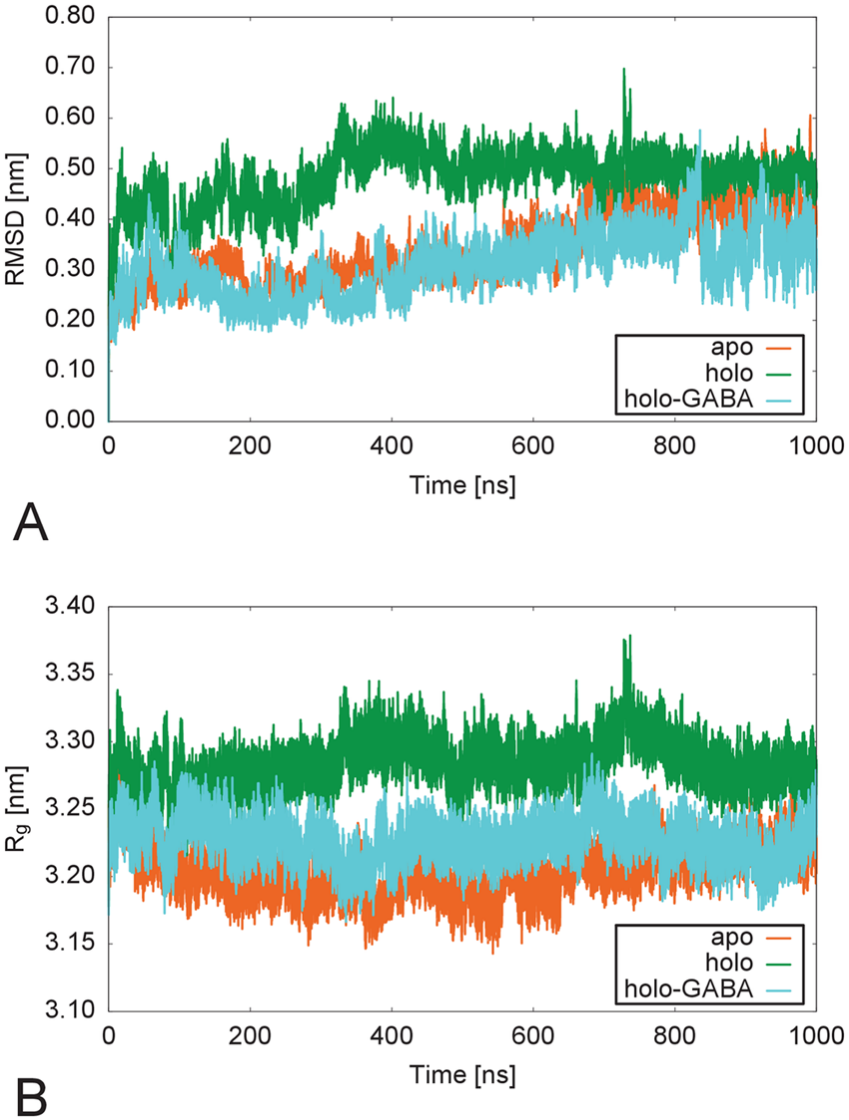
Backbone RMSD and Radius of gyration. Comparison of (**A**) the backbone RMSDs and (**B**) Radii of gyration (R_g_) calculated over the 1 μs simulation for the three GabR form dimers. Color code is reported in the plot inset. Plot created with the software gnuplot v. 4.4 (http://www.gnuplot.info/).

**Figure 3:**
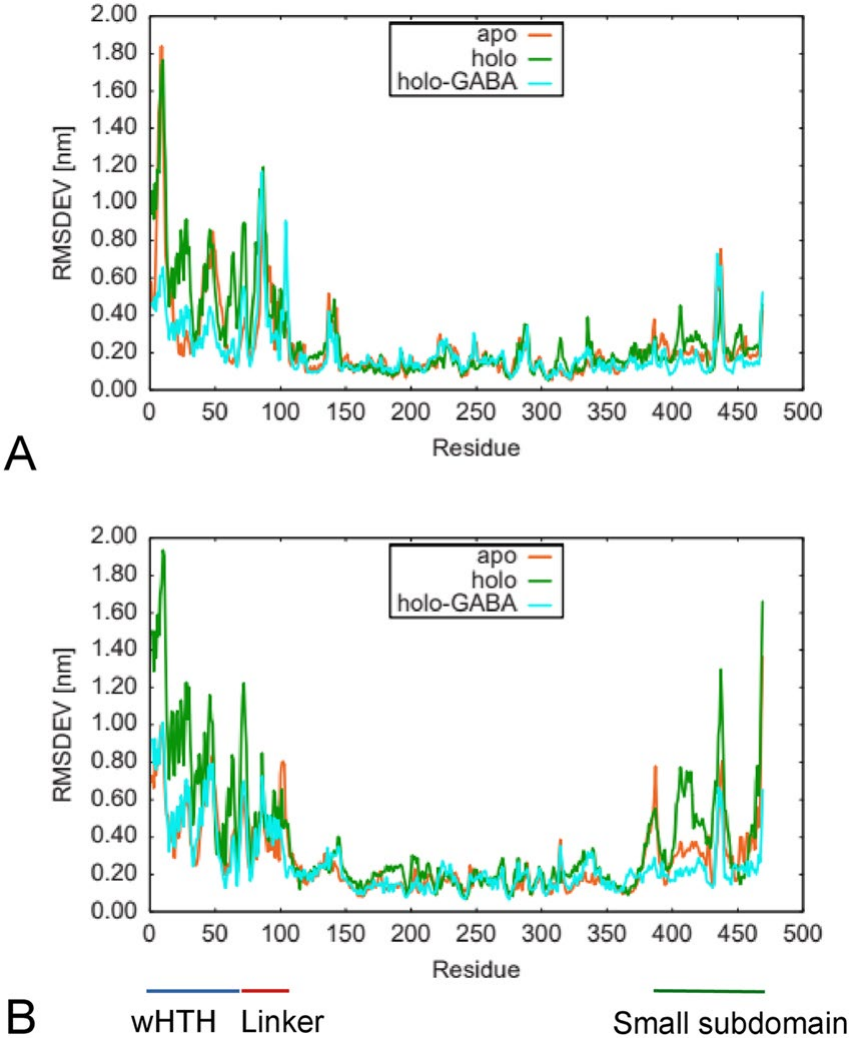
Comparison of the residue-wise RMSD of the three GabR forms. Plot of the residue-wise RMSD calculated over the 1 μs simulation for the three GabR forms. Chains A and B are reported in the upper (**A**) and lower (**B**) chart, respectively. Color code is reported in the plot inset. Lines beneath the lower chart mark the positions of the GabR regions specified by labels. Plot created with the software gnuplot v. 4.4 (http://www.gnuplot.info/).

The three GabR forms appear stable over the entire 1 μs simulation period (Fig. 2). The backbone RMSDs (Root Mean Square Deviation) charted in Fig. 2A show that overall, holo form is more mobile than holo-GABA and apo forms. Chain B appears always more mobile than chain A in all the three forms (Supplementary Fig. S3). Accordingly, holo GabR radius of gyration (Fig. 2B) appears constantly higher than in the other forms. To localize the structural subsets of GabR that more contribute to the fluctuations, the residue-wise RMSD has been calculated and charted (Fig. 3). The plot suggests that the HTH and linker regions are the most fluctuating parts in all the three forms. However, holo and holo-GABA forms are the most and the least flexible, respectively, whereas the apo GabR is positioned at an intermediate level. Interestingly, N- and C-terminal segments tend to be more mobile than the middle portions of the molecule. Noteworthy, the residue-wise RMSD suggests that the C-terminal portion of the chain B, corresponding to the small AAT subdomain (residue interval 370–469), is most mobile in the holo GabR and least mobile in holo-GABA form. The apo form subdomain displays in-between mobility. RMSF (Root Mean Square Fluctuation) plots (Supplementary Fig. S4) are in line with the indications of the RMSD analyses.

### Essential dynamics

**Figure 4:**
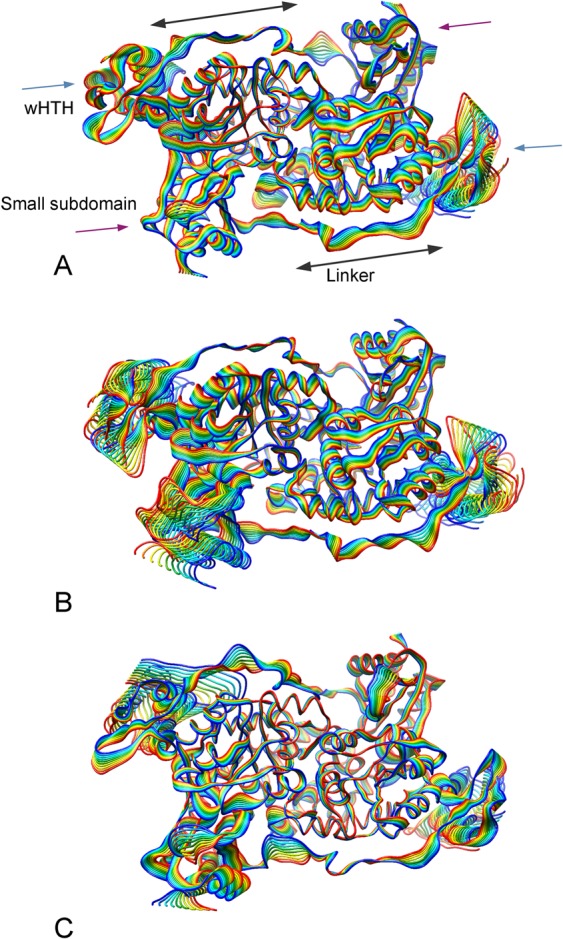
Projections over the first eigenvector. Projection of the MD trajectories on the first eigenvector of (**A**) apo GabR, (**B**) holo GabR and (**C**) holo-GABA GabR. Color gradient from blu to red indicates increasing simulation time. Frames have been sampled every 100 ns. All the three GabR structures are oriented approximately as in Fig. 1. In (**A**), wHTH domains, linkers and small subdomains in each subunit are demarked by labelled blu, grey and magenta arrows, respectively. This figure has been obtained with the program UCSF Chimera v 1.13 (http://www.cgl.ucsf.edu/chimera)^56^.

To capture and visualize the dominant motions in the three GabR forms, the essential dynamics technique was applied. Comparisons of the projections of the motions onto the first eigenvector for the three GabR forms are displayed in Fig. 4. Qualitatively, similarities among the three trajectories appears evident. In particular, the HTH domains fluctuate over the AAT domain surface, even though along different orientations in the three cases. Likewise, in all cases linkers display some flexibility. However, the small subdomain of the subunit B AAT domain of the holo GabR form (Fig. 4B) is more mobile than in the corresponding holo-GABA form (Fig. 4C). The small subdomain of apo GabR appears to be the least mobile (Fig. 4A). This pattern can be symptomatic of the stiffening of the small domain with respect of the large domain within the AAT subunit, induced by GABA binding.

**Figure 5:**
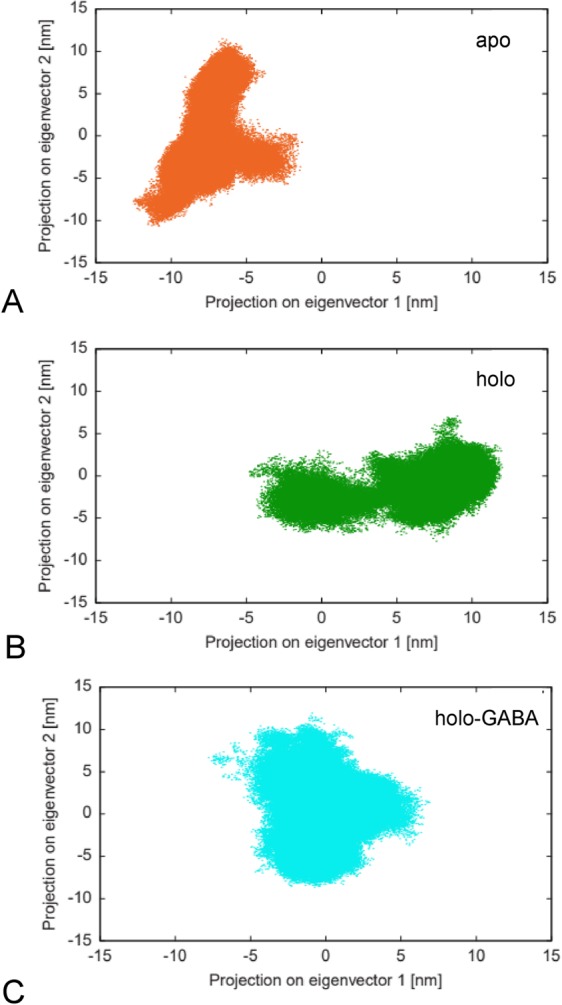
Projections over the first two eigenvectors. Projection of the Cα carbon MD trajectories of the three forms of GabR onto the first two eigenvectors of the same subspace calculated over the pooled trajectories. (**A**) apo, (**B**) holo and (**C**) holo-GABA. Colors label the three GabR forms as reported in the preceding Figures. Plot created with the software gnuplot v. 4.4 (http://www.gnuplot.info/).

Projection of trajectories onto the two-dimensional plane defined by the first and the second eigenvectors of the concatenated trajectories provides further evidence that the holo GabR dynamics differs from that of apo and holo-GABA forms (Fig. 5). In particular, the trajectory projections of the holo-GABA form are evenly distributed. On the contrary, projections of the holo and apo form appears to be distributed in at least two clusters. This pattern may be generated by the fluctuation of holo GabR between two main conformations.

### HTH and AAT domains

Surface contacts between the AAT and HTH domains have been monitored during the simulation in the three GabR forms (Supplementary Fig. S5) using the distance threshold value of 0.3 nm which allows to intercept also the H-bonds in addition to other short-range interactions. Overall, the number of contacts between the HTH and AAT domains follow these trends: the number of contacts between subunit B HTH and subunit A AAT domains are higher in the holo-GABA followed by apo and holo forms; on the contrary, the symmetry-related contacts are more numerous in the apo, followed by the holo and holo-GABA forms. These observations suggest the presence of a possible asymmetrical pattern of contacts between the two HTH-AAT domain interfaces which is at this stage difficult to justify in structural terms.

The number of contacts between the two AAT domains is greater in the apo form, followed by the holo-GABA and the holo form. It may be argued that the AAT-domain interface is most and least compact in the apo and holo form, respectively while holo-GABA sits at an intermediate level.

**Figure 6:**
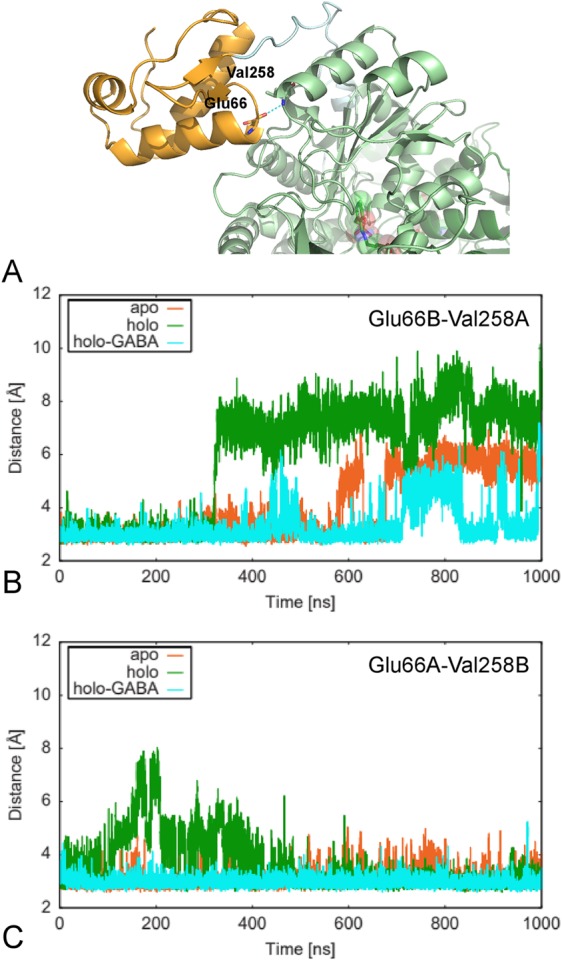
Distance monitoring of the H-bond connecting two helices. (**A**) GabR structure in correspondence of HTH and AAT interface. The H-bond connecting the helices from subunit B HTH and subunit A AAT domains is shown. Distances between the carbonyl oxygen of the residue Glu66B and the peptide nitrogen of the residue Val258A (**B**) and Glu66A and Val258B (**C**) monitored over the 1 μs of simulation time. Figure in panel **A** has been drawn with the software open-source PyMOL v. 1.8.4.0 (https://pymol.org/)^55^. (**B,C**) Plots were created with the software gnuplot v. 4.4 (http://www.gnuplot.info/).

A more detailed comparison of the contacts between the HTH and the AAT domain surfaces has been carried out by monitoring the distance between the carbonyl oxygen of the residue Glu66 and the peptide nitrogen of the residue Val258. Glu66 is the C-terminal residue of the α-helix encompassed by positions 53–66 in the HTH domain while Val258 is the N-terminal residue of the α-helix delimited by positions 258–268 in the AAT domain of the other subunit (Fig. 6A). The two atoms interact via a hydrogen bond that contributes to maintain the axis alignment of the helices from the two domains.

Comparison of the distance variations between the two interacting atoms in the three GabR forms (Fig. 6B,C) indicates that they tend to drift apart in the apo and holo forms with a more pronounced tendency in the latter. The same interaction is more stable in the holo-GABA form. Moreover, the differences are clearer in the pair subunit B HTH – subunit A AAT domains according to what reported above for the overall HTH-AAT interface.

### Active site

**Figure 7:**
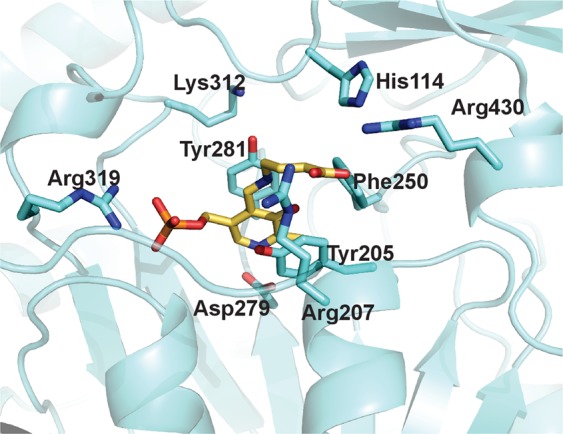
Active site. Active site of the chain B of the holo-GABA GabR form. Side chains discussed in the text are displayed as stick models and labelled accordingly. Stick PLP-GABA complex is coloured in yellow. This figure has been created with the software open-source PyMOL v. 1.8.4.0 (https://pymol.org/)^55^.

**Figure 8:**
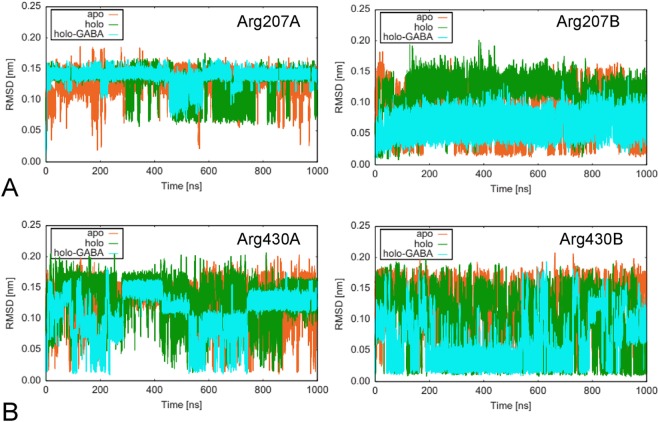
Residue RMSD. RMSD plot of the residues Arg207 (**A**) and Arg430 (**B**). Chain A and B are reported in the left and right charts, respectively. Plot created with the software gnuplot v. 4.4 (http://www.gnuplot.info/).

**Figure 9:**
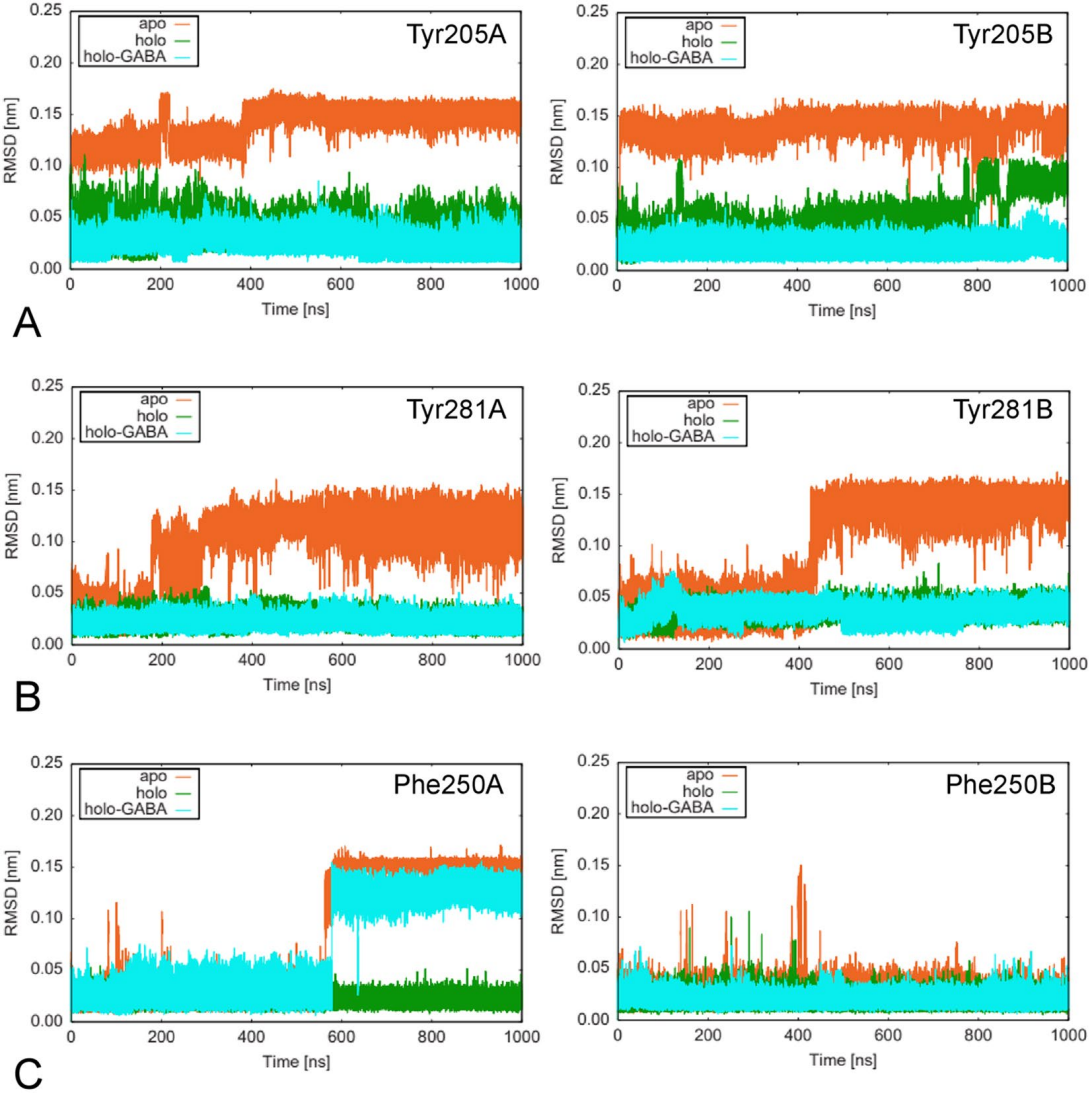
Residue RMSD. RMSD plot of the residues (**A**) Tyr205, (**B**) Tyr281 and (**C**) Phe250. Chains A and B are reported in the left and right charts, respectively. Plot has been created with the software gnuplot v. 4.4 (http://www.gnuplot.info/).

Focus has been given to the comparison of changes occurring at the active site in the three GabR structures during MD simulations (Fig. 7). The dynamics trajectories of the residues in contact with PLP (holo) and PLP-GABA (holo-GABA) have been monitored (Table 2). From this analysis, it appears that residues interacting with GABA carboxylate, Arg207 (Fig. 8A) and Arg430 (Fig. 8B), are consistently stiffer in both subunits of holo-GABA form if contrasted with the other two forms. The two residues are restrained by the H-bonds formed with the substrate carboxylate. Arg430 belongs to the small subdomain. Tyr281, stacking with the PLP ring, appears substantially constrained in the holo and holo-GABA forms. Indeed, comparison of the active sites of the three forms shows that Tyr281 is flipped in the apo GabR structure (Fig. 9B). Tyr205 is the other residue of the aromatic sandwich that interacts with the PLP pyridine ring. In this case also, Tyr205 of both chains appears less mobile (Fig. 9A) in holo-GABA GabR and more fluctuating in the apo form. The last residue of the “aromatic cage” is Phe250. The side chain of this residue is rather stiff in chain B of the three forms while it undergoes a conformation transition at about 600 ns in the sole chain A of apo and holo-GABA GabR forms (Fig. 9C). Arg319 possibly interacting with the PLP phosphate group appears to be rather stiff in all the forms especially in chain A (Supplementary Fig. S6). The remaining active site residues shown in Table 2, namely His114, Asp279 and Arg430 display a similar dynamics pattern in the three GabR forms.

**Table 2:** Residues at the GabR active site involved in ligand interaction.

Residue	Role
His114	Interaction with GABA carboxylate
Tyr205	Stacking with PLP ring
Arg207	Interaction with GABA carboxylate
Phe250	Interaction with the phenolate side of PLP ring
Asp279	Interaction with pyridine nitrogen
Tyr281	Stacking with PLP ring
Lys312	Internal aldimine formation with PLP
Arg319	Interaction with PLP phosphate group
Arg430	Interaction with GABA carboxylate

### Linker

**Figure 10:**
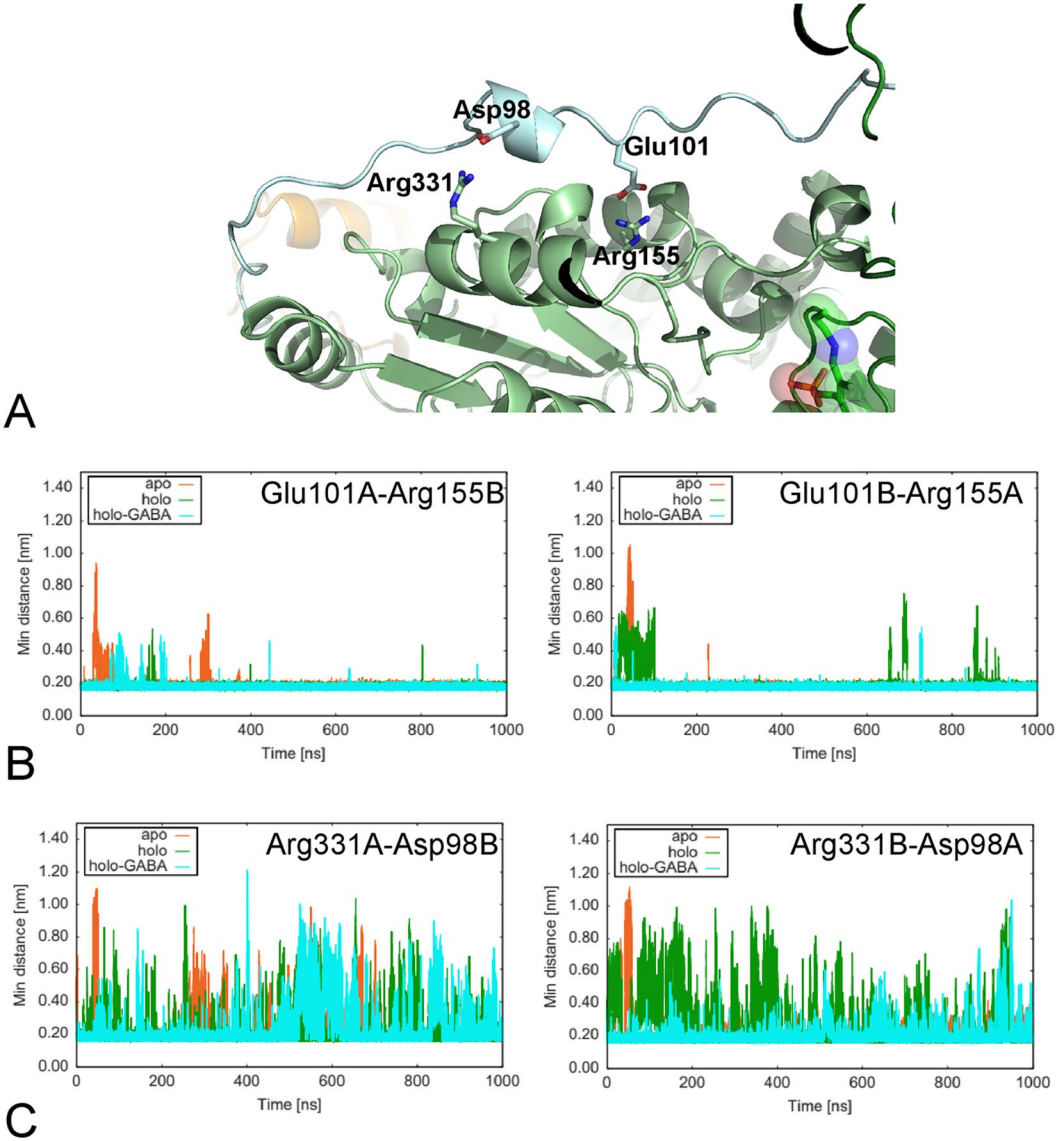
Linker. (**A**) Salt bridges at the interface between the chain A Linker and the chain B AAT domain. Colors are as in Fig. 1. Residues involved in the discussed salt bridges are depicted with stick models and labelled. (**B**) Minimum distance between Glu101A and Arg155B (left panel) and vice versa (right panel). (**C**) Minimum distance between Arg331A and Asp98B (left) and vice versa (right). Figure in panel **A** has been drawn with the software open-source PyMOL v. 1.8.4.0 (https://pymol.org/)^55^. (**B**,**C**) Plots have been created with the software gnuplot v. 4.4 (http://www.gnuplot.info/).

Contacts between linker and AAT domain have been previously analyzed during shorter molecular dynamics experiments^32^. Particular attention has been focused onto the salt-bridge interactions between residues Glu101 and Asp98 of the linker and Arg155 and Arg331 of the AAT domain of the other subunit, respectively (Fig. 10A). These residues are in a position that resembles a hinge around which linker fluctuates. Salt bridges have been monitored during the simulation by measuring the minimum distance between the partner residues. The interaction Glu101-Arg155 between chains A and B appears stable with all the three GabR forms for the entire duration of the MD, except for the first 100 ns (Fig. 10B). Within this time frame, a fluctuation of the distances can be seen in the apo and holo forms. The distance Asp98B-Arg331A fluctuates in a similar way in the three forms and shows peaks of about 1 nm (Fig. 10C). Distance Asp98A–Arg331B in the symmetry related chain B linker (Fig. 10C, left panel) appears more stable in apo and holo-GABA forms than in holo GabR.

### Correlation and protein structure network analysis

Protein Structure Network (PSN) analysis is a powerful approach to unveil dynamics correlations among different portions of the protein structure. In our case PSN has been utilized as a comparative tool to study the overall effect of the presence (or absence) of PLP and PLP-GABA adduct at the active site on the GabR dynamics. The elements of the Dynamic Correlation Matrix range from −1 (the residues are anti-correlated, they move in the same period but in opposite phase) to +1 (the residues are correlated, they move in the same period and phase). The number of correlations between −1.0 and −0.2 is almost the same for the apo and holo GabR forms (about 97,000), whereas holo-GABA has fewer negative correlations (about 85,000). The number of correlations ranging from +0.2 to +1.0 is similar in the holo and holo-GABA forms (112,000 and 117,000, respectively) while is higher in the apo form (nearly 137000). The high occurrence of negative correlations in holo GabR can possibly suggest that this structure is the least compact among the three. On the contrary, the lowest number of total and negative correlations in the holo-GABA forms reveals that this molecule is less prone to expand and deviate from its initial configuration (Supplementary Fig. S7).

**Figure 11:**
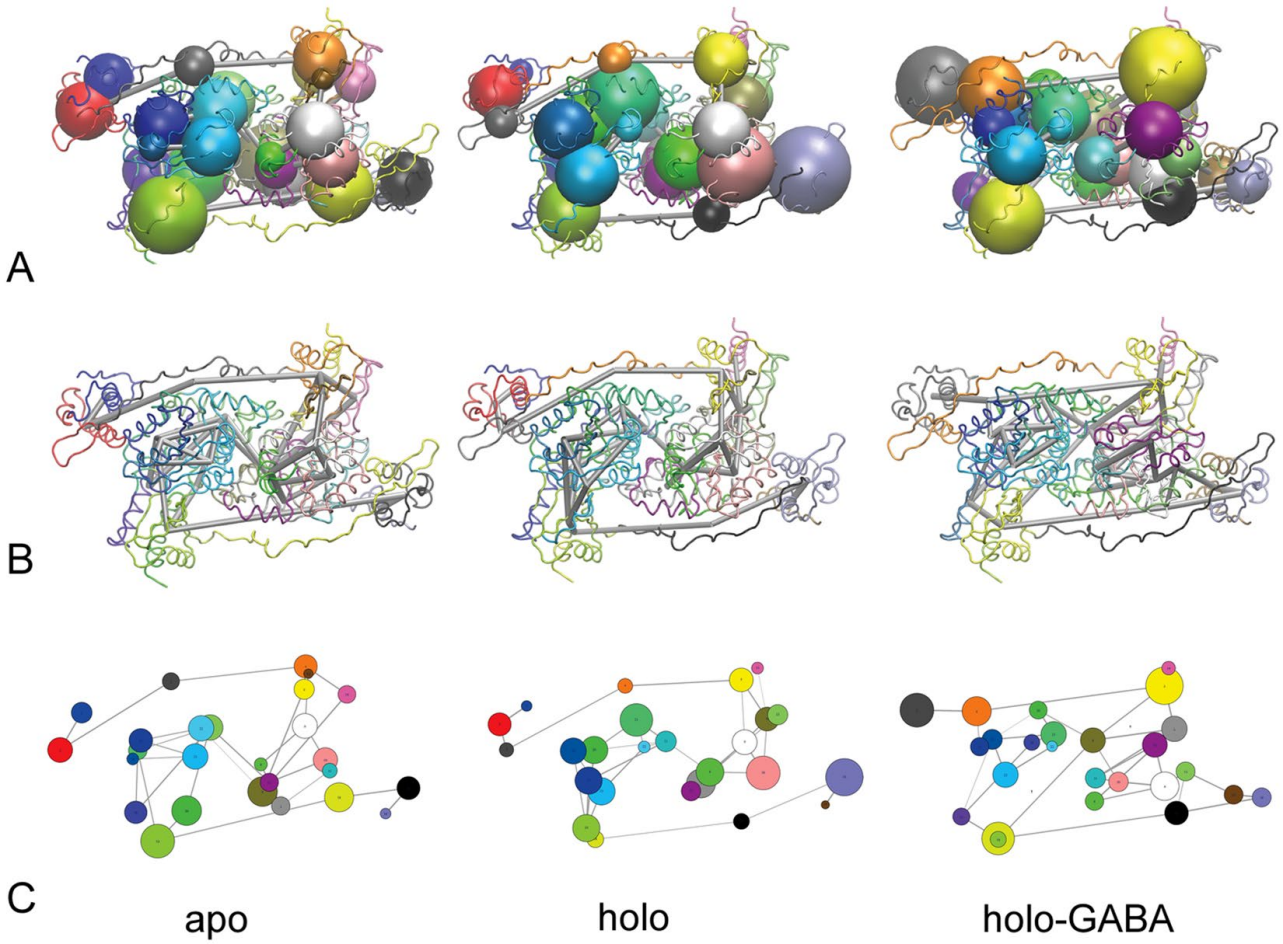
Protein Structure Networks. Correlation network analysis for apo, holo and holo-GABA forms. Different representations are reported. (**A**) Spheres indicate the residue communities distinguished by different colors superposed to the GabR structure displayed as a grey ribbon. Sphere radius is proportional to the community dimension. Grey tubes connect highly correlated communities. (**B**) Same as A without community spheres to highlight the different pattern of correlations. (**C**) Simplified two-dimensional map. Circles are equivalent to the community spheres and connecting grey lines to the tubes. Structures are oriented approximately as in Fig. 1. Panel A and B were obtained with the program VMD v. 1.9.3 (https://www.ks.uiuc.edu/Research/vmd/vmd-1.9.3/)^54^. Figure in panel C was created with Bio3D^58^.

Setting network parameters d_cut_ = 5 Å, c_cut_ = 0 and p_cut_ = 0.75, 26 communities with member counts between 16 and 57 can be found in the apo form; 25 communities with a number of members between 14 and 63 in the holo form, and 27 communities containing between 18 and 63 members in the holo-GABA GabR (Fig. 11). Communities of the HTH and AAT domains of the other subunit tend to be uncorrelated in the apo and holo GabR forms. On the contrary, in holo-GABA GabR the HTH and AAT domains appear to be significantly correlated (Fig. 11). The linker seems to play a less important role in the holo-GABA as a mediator of the interaction between the HTH and AAT domains. Overall, residues within the AAT domain of the holo-GABA form appear more densely correlated than in the other two forms as a possible consequence of higher compactness and stiffness.

In the framework of PSN, the betweenness metric is used to measure the importance of the nodes in the network. Thus, residues with a high betweenness are candidate to play a functional role in a protein. After normalization and setting a threshold value equal to 0.6, some general trends can be noted (Supplementary Fig. S8). The apo has the lowest number of high betweenness nodes, the holo the highest number. The same is true for the betweenness values. Notably the group of chain A residues with positions ranging from 317 to 323, comprising Arg319, have high betweenness in all the three networks. Other groups found in chain B of holo-GABA and especially in both chains of the holo form have no intersection with each other (Supplementary Fig. S8).

## Discussion

In bacteria, expression of many genes is subject to variation in response to environmental changes. Such regulation determines the overall fitness of the bacterial cell. Within this context, transcription regulators play a crucial role in physiological adaptation by controlling gene expression through interference with binding of RNA polymerase to DNA. The activity of the regulators can be influenced by the interaction with effector molecules or ions, by physical parameters (temperature or pH), and protein-protein interaction or protein modification. So far, very little is known about the detailed molecular mechanisms underlying the allosteric modulation caused by effector binding on the TF functional properties.

In this work we have been focusing on the GabR^25^ a transcriptional regulator belonging to the MocR family, a newly discovered group of regulators structurally related to the fold-type I PLP-dependent enzymes^1^. In *Bacillus subtilis*, this regulator can activate transcription of genes involved in GABA degradation in response to binding of PLP and GABA as an external aldimine adduct^27,29^. To study the structural alterations induced by the presence (or absence) of PLP or PLP-GABA adduct at the GabR active site, an extensive comparative molecular dynamics approach at 1 μs scale has been applied. Therefore, we have been comparing the dynamics of GabR without any ligand (the apo form), of GabR binding PLP (holo), and the holo-GABA form, complexed with the external aldimine PLP-GABA. In this context, the apo GabR form should be considered a reference structure to which compare the properties of the holo and holo-GABA forms. Although it has been demonstrated that *in vitro* the apo form can bind DNA^25,30^, it is not clear whether it exists *in vivo* and whether it has a significant physiological role within the bacterial cell.

Several fold type-I enzymes undergo a transition from a so-called open to a closed form upon substrate binding. The transition has been clearly demonstrated in the case of the enzyme aspartate aminotransferase^7^. The conformation transition consists of the closure, within the single AAT subunit, of the large and small subdomains^36^, this closure is considered essential for an effective and specific catalysis. An analogous movement has been supposed on the basis of examination of the crystallographic structure of the AAT domain^27,29^ of holo-GABA GabR. This conformational change is deemed to be an element of the response to GABA binding and the consequent transcription activation. To this respect, it should be emphasized that the MD experiments described in this work started with initial structures “modelled” onto the holo-GABA GabR AAT domain in “closed” conformation corresponding to the coordinate set 5T4J. Therefore, the dynamics trajectories that have been studied should reflect the propensity of the AAT domain to change from the closed to the open conformation when GabR is in the apo or holo-forms. On the contrary, the holo-GABA form should be already in the closed conformation and should show, as indeed happens, relatively lower mobility and higher stiffness. The results here presented support this view.

Examination of the overall dynamics suggested that the holo GabR form appears rather flexible while the holo-GABA complex is stiffened and more compact, as expected for a closed conformation. Moreover, residue-wise RMSD and RMSF analyses suggest that the most flexible portions in holo GabR are the HTH domain, the linker and the small subdomain. This is particularly evident in the subunit B. Apo GabR sits somewhat at an intermediate level. It should be reminded that apo GabR, like holo, is able to autorepress *in vitro* the expression of its gene but it cannot trigger expression of *gabTD* genome region. Comparison of R_g_ indicates that holo GabR is less compact than holo-GABA form. Increase of compactness of holo-GABA GabR is reflected also by the higher number of contacts between the AAT domains with respect to the holo form. Analysis of the dynamics properties of residues interacting with PLP and GABA suggested once more that the presence of the PLP-GABA external aldimine makes the active-site side chains less flexible. This is particularly evident for Arg207 and Arg430 that interact to the GABA carboxylate. Arg430 is particularly interesting because belongs to the small subdomain and may contribute to the transition open-to-closed conformation. The residues in contact with the PLP have also their flexibility altered. The residues of the “aromatic cage” Tyr205, 281 and Phe250 are more mobile in the apo form, especially Tyr281. However, their flexibility is decreased when interacting to the pyridine ring and it is even more constrained with the presence of GABA. An exception is Tyr281 which undergoes a rotation around the C_α_-C_β_ bond at about 500 ns of dynamics. The dynamics simulation was able to replicate the torsion observed by X-ray crystallography^27,29^ in the external aldimine form of GabR. Tyr281 is indeed believed to prevent interaction of Lys312 with the PLP-GABA Schiff base. Its side chain rotation blocks reformation of the internal aldimine as well as further transamination-like catalysis.

Essential dynamics analysis suggests that the presence of GABA external aldimine changes the distribution and orientation of the principal conformational movements. This redistribution involves mainly the small AAT subdomain and the HTH and linker domains. In general, it may be argued that the movement distribution in holo GabR tends to cluster into two conformational spaces, as shown in the 2D trajectory projections, which may indicate the fluctuation between the open and closed conformation. Holo-GABA GabR displays instead an isotropic distribution of its dynamics. Interesting insights into the global dynamics rearrangements are provided by the PSN analysis. The analysis suggests that in the holo-GABA form the dynamics of the residues in the HTH domains tend to be correlated to those in the AAT domains. Moreover, this evidence is compatible with the presence of a closed, more compact GabR conformation when GABA is bound at the active site. Indeed, this effect is absent in the case of the other two forms. Moreover, the correlation analysis highlighted a different frequency and distribution of positive and negative correlations within the three GabR forms. The high occurrence of negative correlations in holo GabR can possibly suggest that this structure is the least compact among the three. On the contrary, the lowest number of negative and total correlations in the holo-GABA forms reveals that this molecule is less prone to expand and deviate from its initial configuration. However, betweenness analysis fails to indicate clearly any group of hub residue distinctive of the three forms. Overall, holo GabR appears to possess more hubs than apo form. Holo-GABA GabR does not display any peculiar pattern. Common trait is the centrality of the Arg319 segment which is difficult to explain at this stage.

The results here presented are coherent with several experimental observations and considerations reported in literature. There is a consensus that GABA binding at the active site of GabR induces a conformational change. Besides early experimental evidences^25^, results of crystallographic^4,28,29^, atomic force microscopy^30^ and X-ray scattering^37^ experiments support the existence of a GABA-induced conformational modification. Unquestionably, binding of GABA converts GabR into an activator of *gabTD* genes^18^. It should be mentioned that in a few experiments it has been observed that binding of GABA to GabR causes an apparent increase of the radius of gyration^30,37^, namely a possible expansion of the molecule. Considerations based on isothermal titration calorimetry suggested that GABA binding to GabR AAT domain induces changes also in the wHTH N-terminal domains^28,38^. Accordingly, MD simulations and PSN analysis hint at a change in the motion correlations between AAT and wHTH domains upon GABA binding. It has also been suggested that the linker connecting the wHTH and AAT domains may be important for conferring ligand specificity. It may work as a lid covering the GabR active site entrance in the closed conformation^28,38^. Our MD suggests that the linker tends to be less flexible in the holo-GABA form and that it has more stable interactions with the cognate AAT domain. On the contrary, the linker is more flexible when GabR does not bind GABA. Moreover, PSN analysis suggests that the linker in holo-GABA is less correlated to the wHTH and AAT domains.

In conclusion, the results here reported depict a molecular mechanism of GabR transcription activation compatible with the assumption of a transition from open-to-close conformation triggered by GABA reaction with active site PLP. In this case, results hints at a transition from closed-to-open conformation in the holo and, less evidently, apo forms. The same results suggest that holo-GABA tends to remain in the close conformation. GABA external aldimine renders the overall GabR structure more compact through interactions with the surrounding environment mediated by the active site residues. Particularly interesting is the coupling/uncoupling of the HTH domain dynamics with the AAT domains which may suggest how the molecular signals are conveyed from the active site to the HTH domains. The flexibility of the linker would allow the AAT domains to change their interaction with the DNA-bound HTH domains and modify accessibility of the RNA polymerase binding site. When GABA binds at the active site, AAT domains become more compact, closed, while tightening their interaction with the HTH domains. This movement may decrease hindering of RNA polymerase action. On the contrary, holo GabR is overall less compact and the AAT domains are more loosely interacting with the HTH domains. Thus, the volume occupied by the regulator is larger and may block RNA polymerase activity.

## Materials and Methods

### Structure modelling

The structure of the entire GabR dimer in its GABA external aldimine form (holo-GABA) has been reconstructed by merging the Protein Data Bank (PDB) structure denoted by the code 5T4J (reporting the AAT-like GabR domains complexed with GABA external aldimine) with the HTH and linker domains extracted from the structure 4N0B (corresponding to GabR PLP internal aldimine) used in a previous MD experiment. Domain merging has been carried out using homology modeling as implemented in the program Modeller v. 9.17^33^. This program builds homology models based on satisfaction of the spatial restraints extracted from the templates. Homology modelling can be utilized to construct chimeric models by assembling together different portions of two or more structural templates. The way the model portions corresponding to different templates sections are combined is determined by the alignment between the model and the template sequences set by the user. In this case, model and template sequences were from the same protein GabR but the templates corresponded to different GabR forms. Ten models have been constructed and, in each case, the final extensive refinement procedure implemented in Modeller has been applied. The model with the best objective function, which measures the extent of satisfaction of spatial restraints, has been selected.

Quality assessment of the chosen model has been carried out with QMEANDisCo^35^ method available in the QMEAN web site^34^. QMEANDisCo is a composite scoring function providing global and local model quality assessment derived from QMEAN terms plus a term taking into consideration the pairwise residue-residue distances. The QMEANDisCo global score is the average per-residue score and ranges in the interval 0 to 1 (best quality).

The structure of GabR internal aldimine (holo) has been modelled from holo-GABA form by removing the external aldimine and converting the GabR Lys312 into internal aldimine. Conformation of internal aldimine has been taken from the coordinate set 4N0B. Apo form has been modeled from the holo-GABA one upon removal of external aldimine. This procedure assures that the simulations and comparisons are carried out on structures prepared using similar conditions.

Sequence alignments have been displayed with the software Jalview v. 2.10.3 (http://www.jalview.org)^39^.

### Molecular dynamics

The three GabR forms have been prepared for simulations as previously reported^32^. The protein residues have been modeled using the AMBER99SB-ILDN force field. Ligands and non-standard residues in the holo an holo-GABA forms have been described by the Generalized Amber Force Field^40^. Partial charges of the atoms composing the ligands and non-standard residues have been calculated with the Restrained Electrostatic Potential Method (RESP)^41^. Optimization and electrostatic potential analysis have been performed with Gaussian 03^42^ at the Hartree-Fock level with the 6−31 G^*^ basis set. Residue Glu 190B has been protonated consistently with what has been done previously^32^. Protonation states of histidines have been chosen by inspection of the surrounding environment in order to maximize locally the number of hydrogen bonds. The analysis has been completed using the PropKa^43^ method as described^32^. Particle Mesh Ewald (PME)^44^ method has been employed for the calculation of long range electrostatic interactions with a grid spacing for the Fast Fourier Transform of 0.12 nm and a short range cutoff of 1.0 nm. A reciprocal grid of 128 × 128 × 128 cells was used with 4^th^ order B-spline interpolation. Neighbour searching has been performed every 10 steps. Bond lengths involving hydrogen atoms have been constrained to a constant value using the LINCS^45^ algorithm with a harmonic potential. Periodic boundary conditions have been set for the three directions.

The system has been solvatated in an octahedral box adding H_2_O molecules using the TIP3P water model^46^. A buffer of 1.6 nm has been foreseen between the outside of the protein and the box. A physiological concentration of 150 mM has been reached by adding 240 Na^+^ ions and 238 Cl^−^ ions (the excess of sodium ions is to compensate for the net negative charge of the protein). The simulated systems contained more than 260000 atoms each.

The apo and holo systems have been energy minimized with 500 steps of the Steepest Descent algorithm^47^ with an energy step size of 0.01 nm. The holo-GABA system has been minimized with the Polak-Ribière Conjugate Gradient method^47^ (179 steps with a Steepest Descent step every 100 Conjugate Gradient steps). After the energy minimization, the equilibration of the solvent has been performed. The positions of the solute heavy atoms have been restrained with a force constant of 1000 kJ mol^−1^ nm^−2^. The equilibration has been carried out in the NVT ensemble for 5 ns. The temperature has been kept constant at T = 298 K by coupling the system with a v-rescale thermostat^48^. Two coupling groups, protein and non-protein, have been chosen for the best accuracy. Equilibrium simulations have been performed also in the NPT ensemble for 5 ns using a Berendsen pressure bath^49^ with pressure P = 1 atm. A production run of 1000 ns has been simulated for each molecule in the NPT ensemble with a time step for the Leap Frog Verlet algorithm^50^ of 2 fs. Avogadro 1.0.3^51^, Gaussian 03^42^ and Amber 12^52^ have been used for the ligands and non-standard residues topology set up. Gromacs 2016.1^53^ suite has been used for simulation and data analysis. Data produced by Gromacs have been plotted with the program gnuplot v. 4.4 (http://www.gnuplot.info/).

### Trajectory and protein structure network analysis

Visual and numerical trajectory analyses have been carried out with the software VMD v. 1.9.3 (https://www.ks.uiuc.edu/Research/vmd/**)**^54^ and the tools of the Gromacs package (http://www.gromacs.org)^53^. Structures have been displayed and analysed with the graphic programs open-source PyMol v. 1.8.4.0 (https://pymol.org)^55^ and UCSF Chimera v. 1.13 (http://www.cgl.ucsf.edu/chimera/)^56^. Ad-hoc Python and Bash scripts were written whenever necessary. The principal component analysis (PCA) has been performed with the Gromacs tool by diagonalizing the covariance matrix of the atomic displacement according to the method proposed^57^. The PCA analysis have been applied to the concatenated trajectories of the three GabR forms to assure eigenvector homogeneity during comparison. The R package Bio3D has been used for Protein Structure Network analysis^58^. Dynamic cross correlation maps^55^ (DCCMs) and protein structure networks (PSNs) have been computed as implemented in Bio3D. Residues communities, namely highly correlated residue subset, have been computed using the Newman and Girvan algorithm^59^. A contact map has been considered to filter the elements of the DCCM based on distance and time persistence cut-offs. A correlation cut-off parameter has been considered to select only significant interactions. Due to the computational burden, the trajectories have been undersampled in order to retain only 1000 frame. Network community analysis depends on different cut-off parameters. The distance (d_cut_) and the persistence (p_cut_) cut-offs are used to filter the DCCM (two residues are in contact if they are closer than the distance cut-off for at least a given number of frames). The network correlation (c_cut_) cut-off, filters on the DCCM coefficients values. The parameters utilized in the work have been set accordingly to the values accepted and reported in literature^60,61^.

## Supplementary information

Supplementary Information

## Data Availability

Trajectory and accessory files necessary to reproduce the results are available upon direct request to the authors.
