# Supplementary material for: Conformational transitions induced by γ-amino butyrate binding in GabR, a bacterial transcriptional regulator
Source: Sci Rep. 2019 Dec 17;9:19319. doi: 10.1038/s41598-019-55581-1 (PMC6917764; doi:10.1038/s41598-019-55581-1)
Supplement: Supplementary file 1 — Supplementary Information [file 41598_2019_55581_MOESM1_ESM.docx]

**Conformational transitions induced by γ-amino butyrate binding in GabR, a bacterial transcriptional regulator**

Mario Frezzini^3^, Leonardo Guidoni^2^ and Stefano Pascarella^1,*^

^1^Department of Biochemical Sciences, Sapienza, University of Rome, 00185 Rome, Italy

^2^Department of Physics and Chemistry Sciences, University of L’Aquila, 67100 L’Aquila, Italy

^3^Department of Information Engineering, Computer Science and Mathematics, University of L’Aquila, 67100 L’Aquila, Italy

*Corresponding author

Stefano Pascarella

Dipartimento di Scienze biochimiche

Università La Sapienza

P.le A. Moro, 5

00185 Roma

Stefano.Pascarella@uniroma1.it


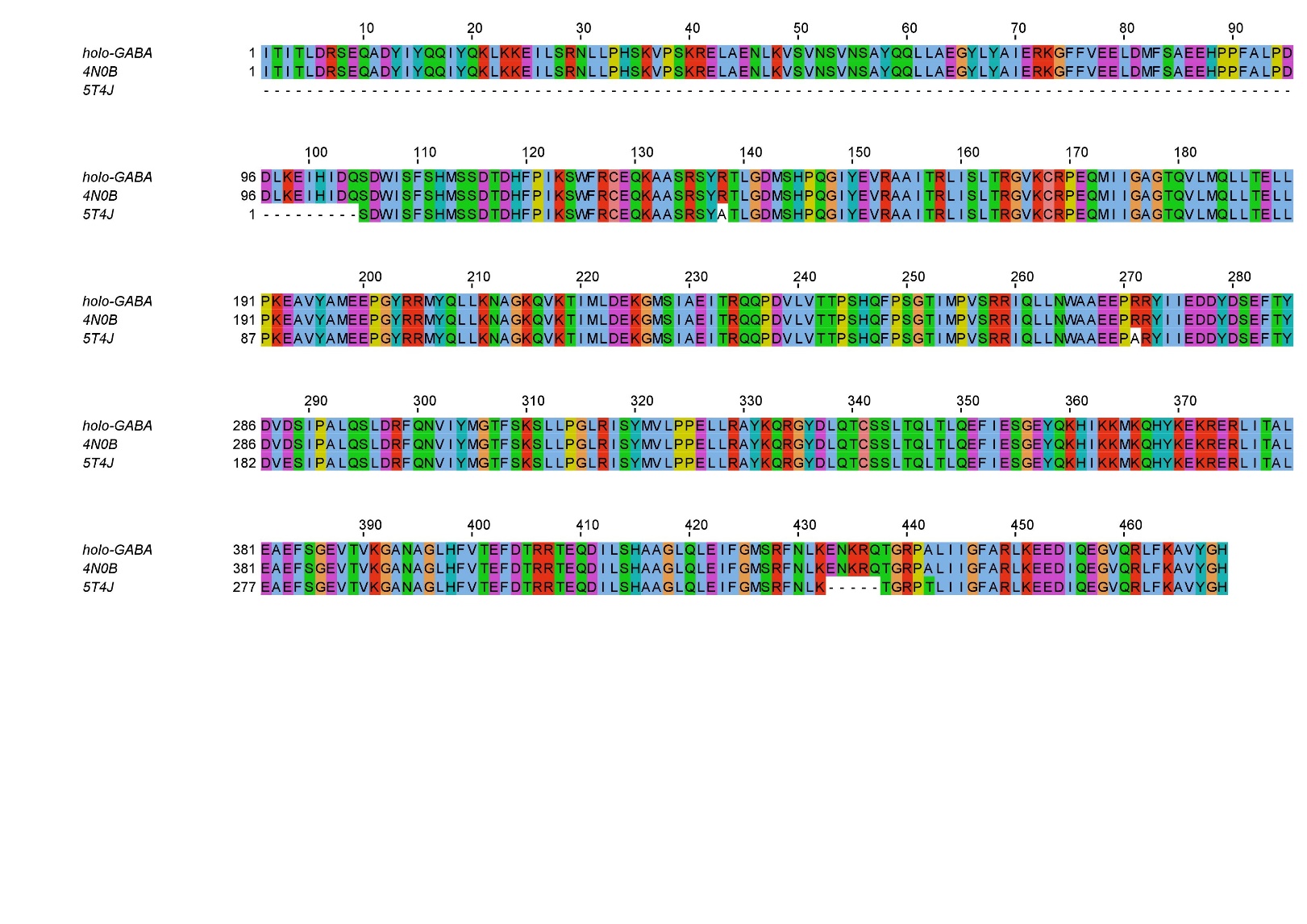


**Supplementary Figure S1. Sequence alignment used for homology modelling**

Sequence alignment among the model holo-GABA GabR form and the sequences of holo (PDB code 4N0B) and external aldimine AAT domain (code 5T4J) forms. ClustalW color scheme indicate residue physico-chemical characteristics. The Figure has been drawn with the program Jalview v. 2.10.3 (http://www.jalview.org).

**
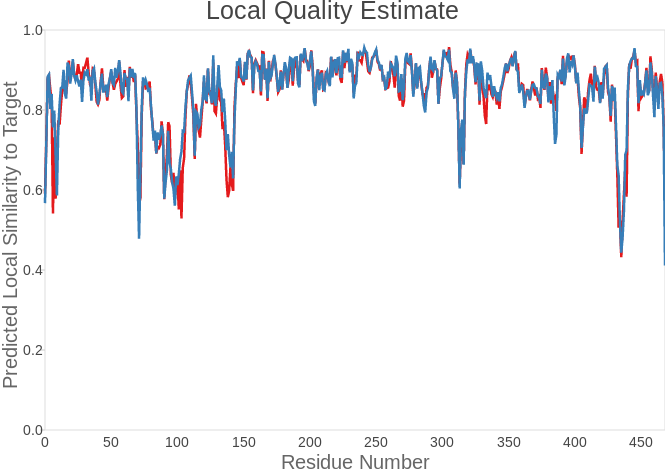
**

A

**
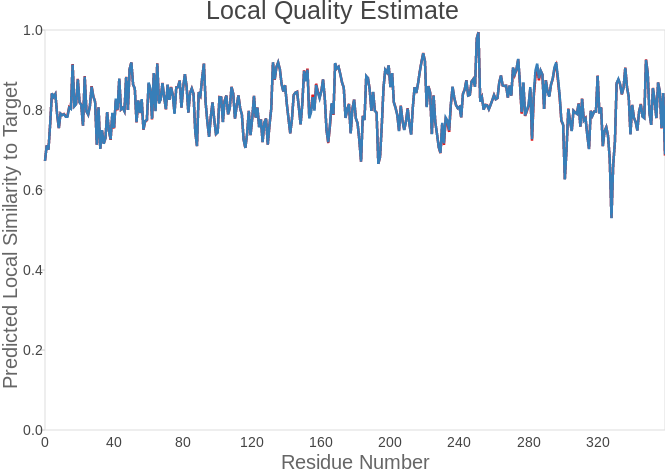
**

B

**Supplementary Figure S2. Local model quality calculated by QMEAN**

Plot of the local quality assessed by the QMEAN score of the holo-GABA GabR form (A panel) and the template 5T4J (B). Red and blu lines refer to chain A and B, respectively. Plots created by the server QMEAN (https://swissmodel.expasy.org/qmean/).

Stefano.Pascarella@uniroma1.it


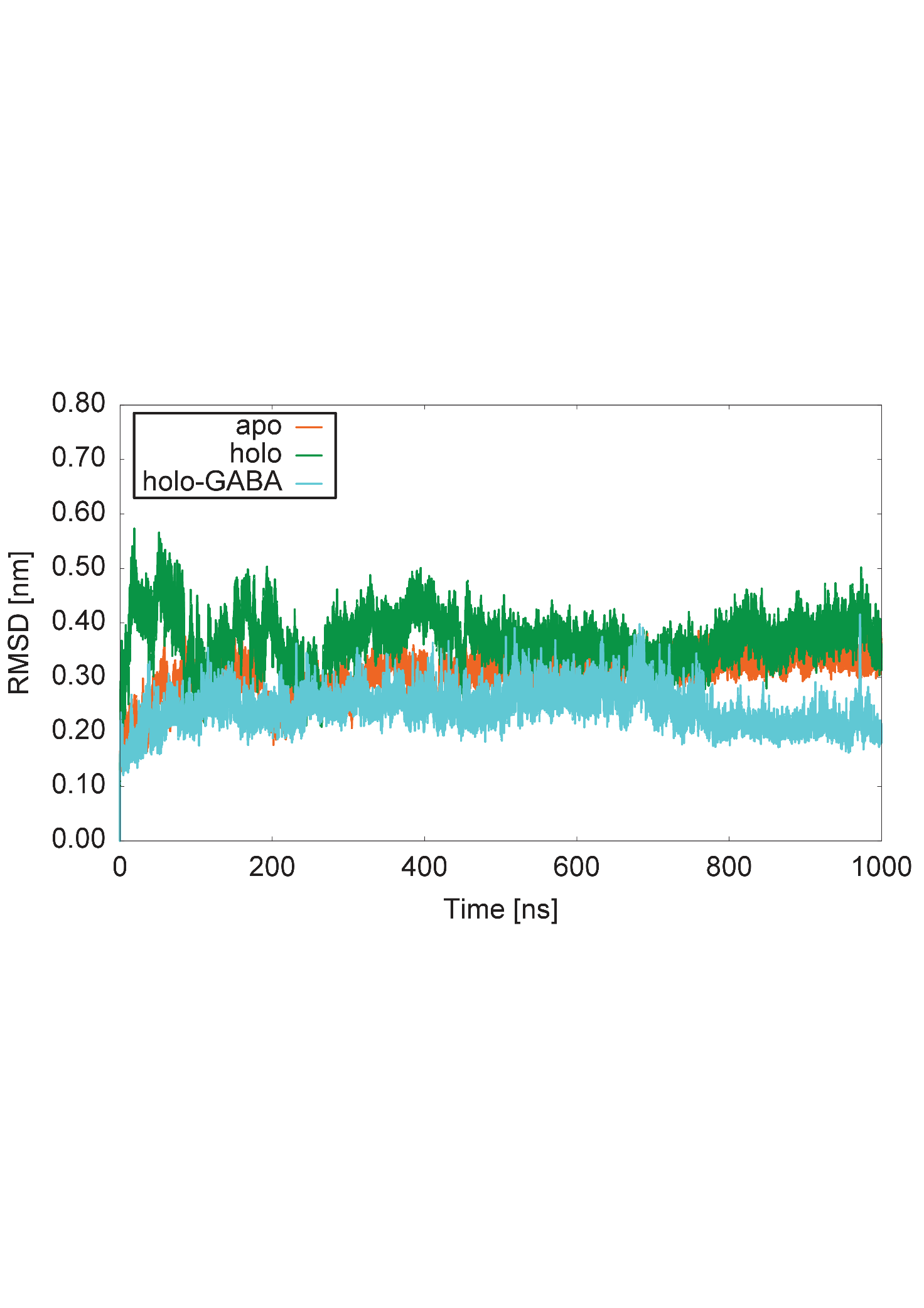


A

**
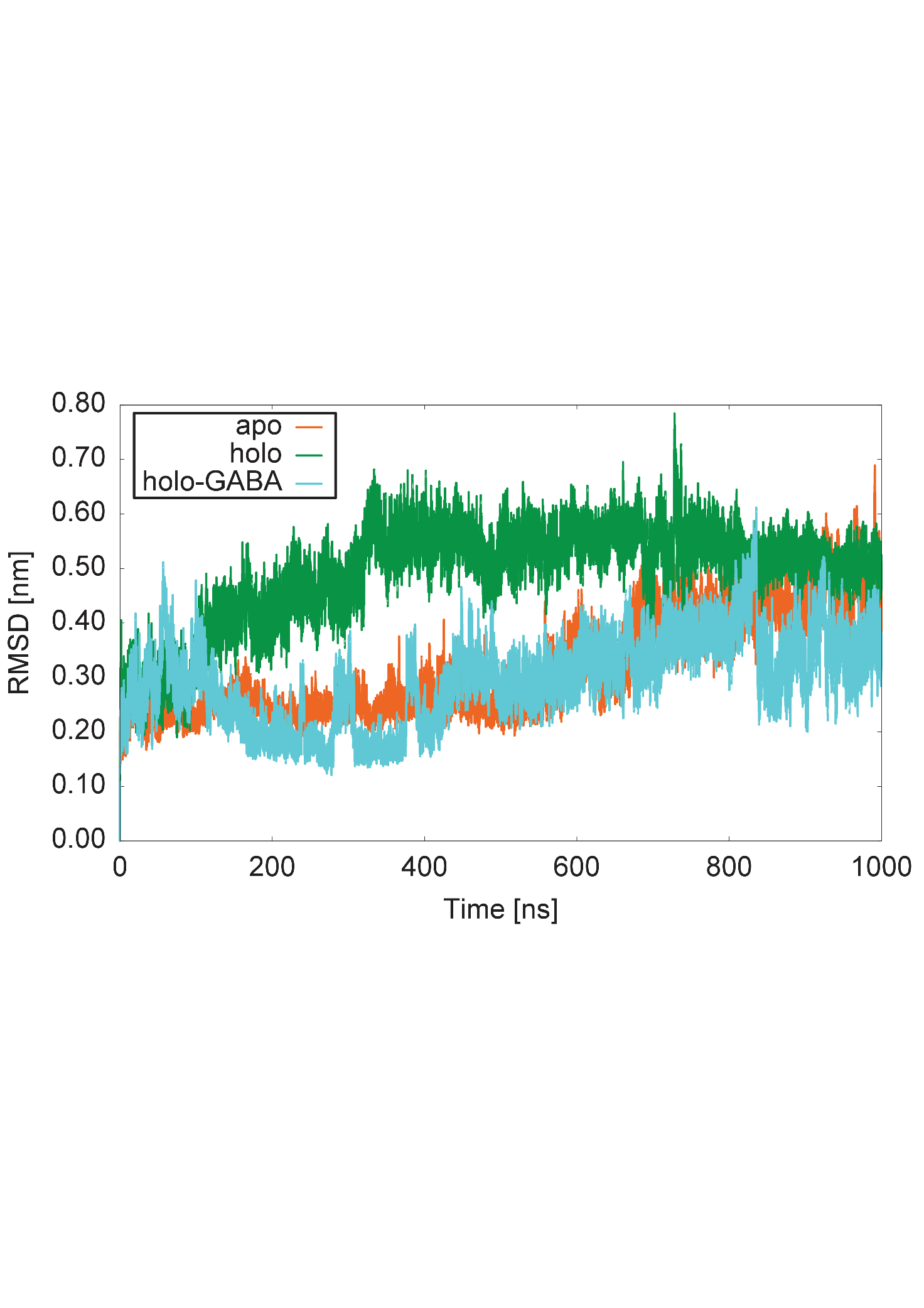
**

B

**Supplementary Figure S3. Backbone RMSD**

Comparison of the backbone RMSDs of the chains A and B of the three GabR forms over 1 μs simulation (A and B panels, respectively). Color code is reported in the plot inset. Plots have been created with the software gnuplot v. 4.4 (http://www.gnuplot.info/).

**
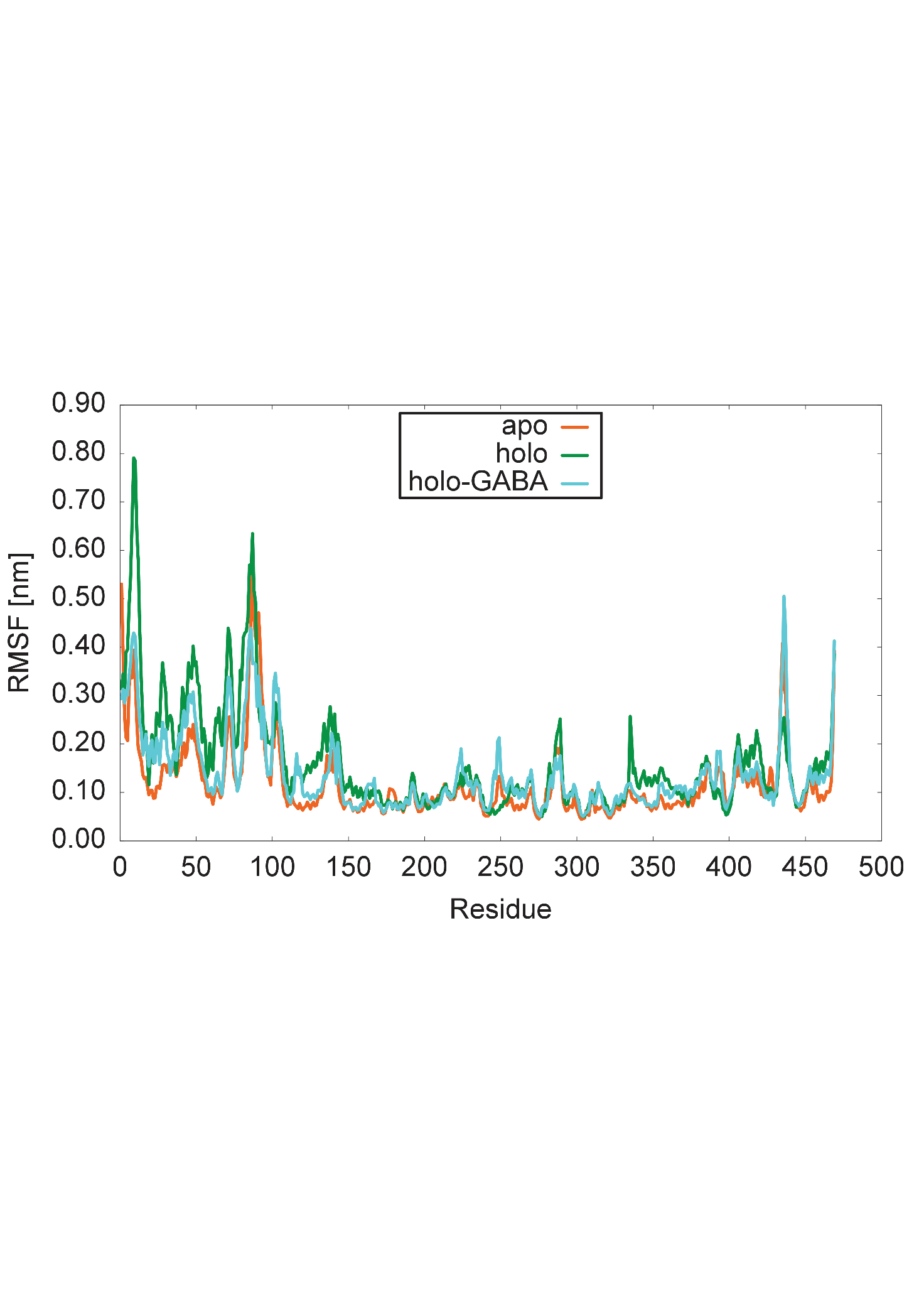
**

A

**
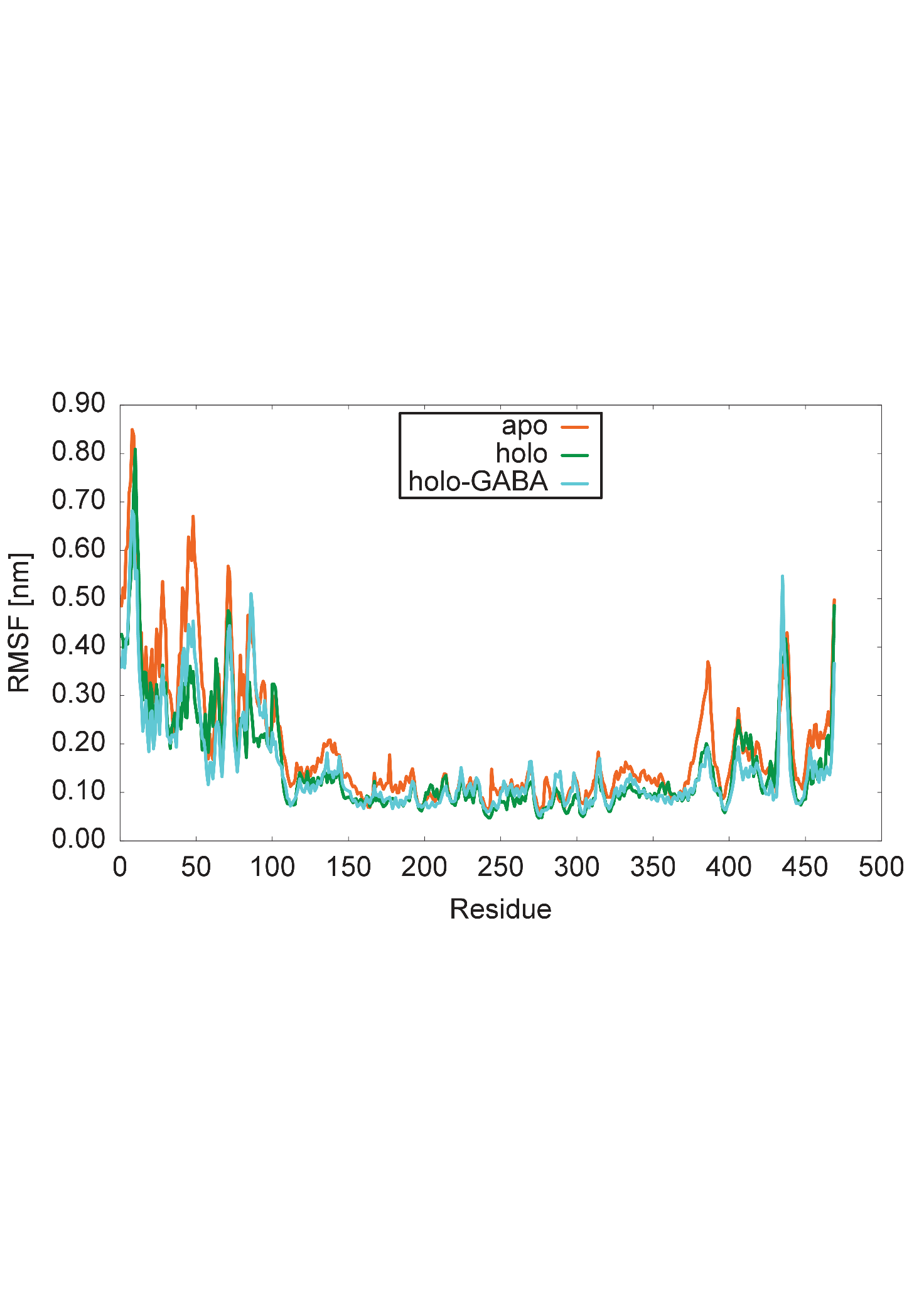
**

B

**Supplementary Figure S4. Residue-wise RMSF**

Comparison of the residue-wise RMSF of chains A and B of the three GabR forms (A and B panels, respectively). Color code is reported in the insets. Plots have been created with the software gnuplot v. 4.4 (http://www.gnuplot.info/).

A

**
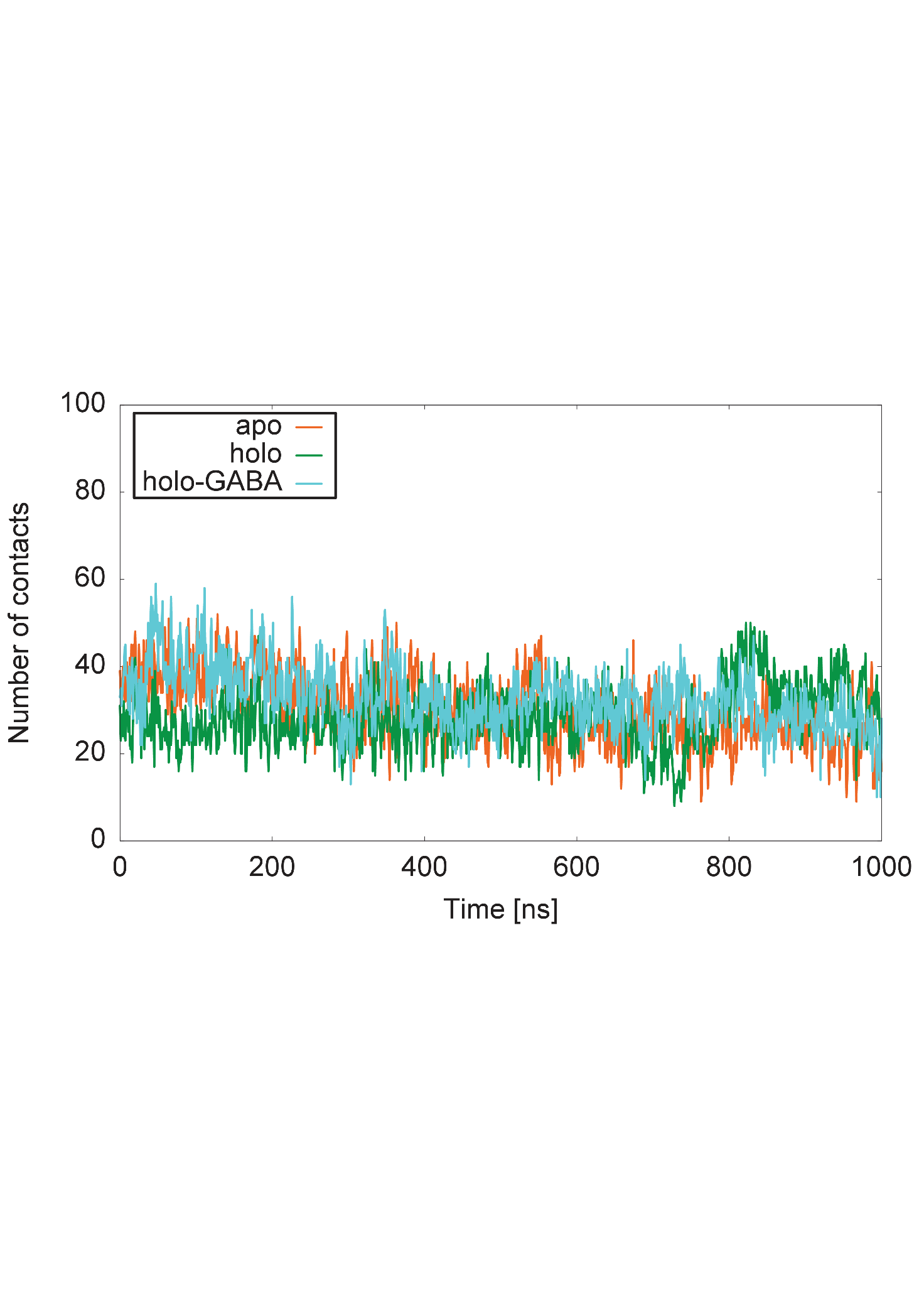
**

A

**
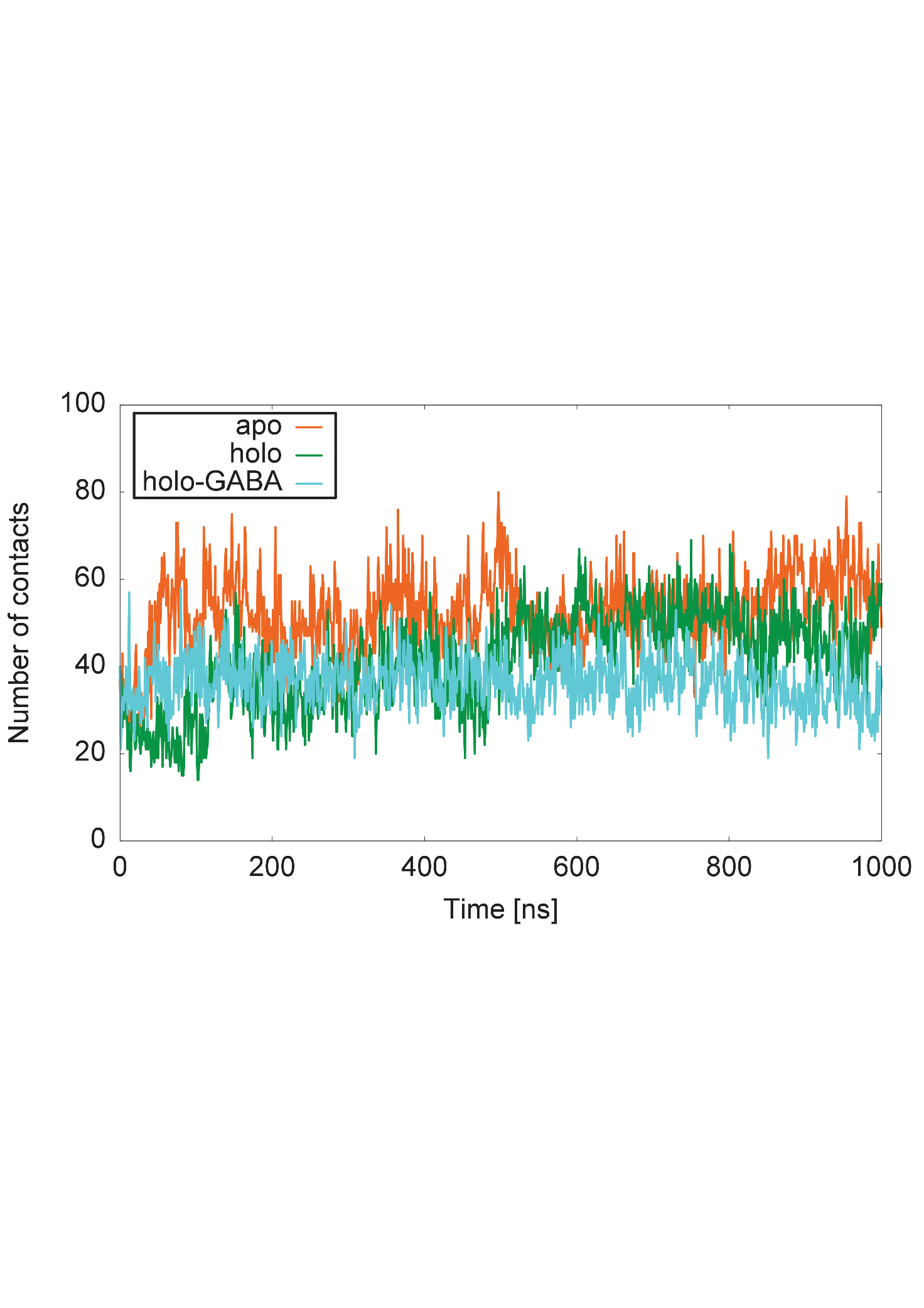
**

B

**Supplementary Figure S5. Number of contacts**

Comparison of the number of contacts between the HTH and AAT domains. A and B panels refer to contacts between chain B HTH - chain A AAT and vice versa, respectively. Plots have been created with the software gnuplot v. 4.4 (http://www.gnuplot.info/).

**
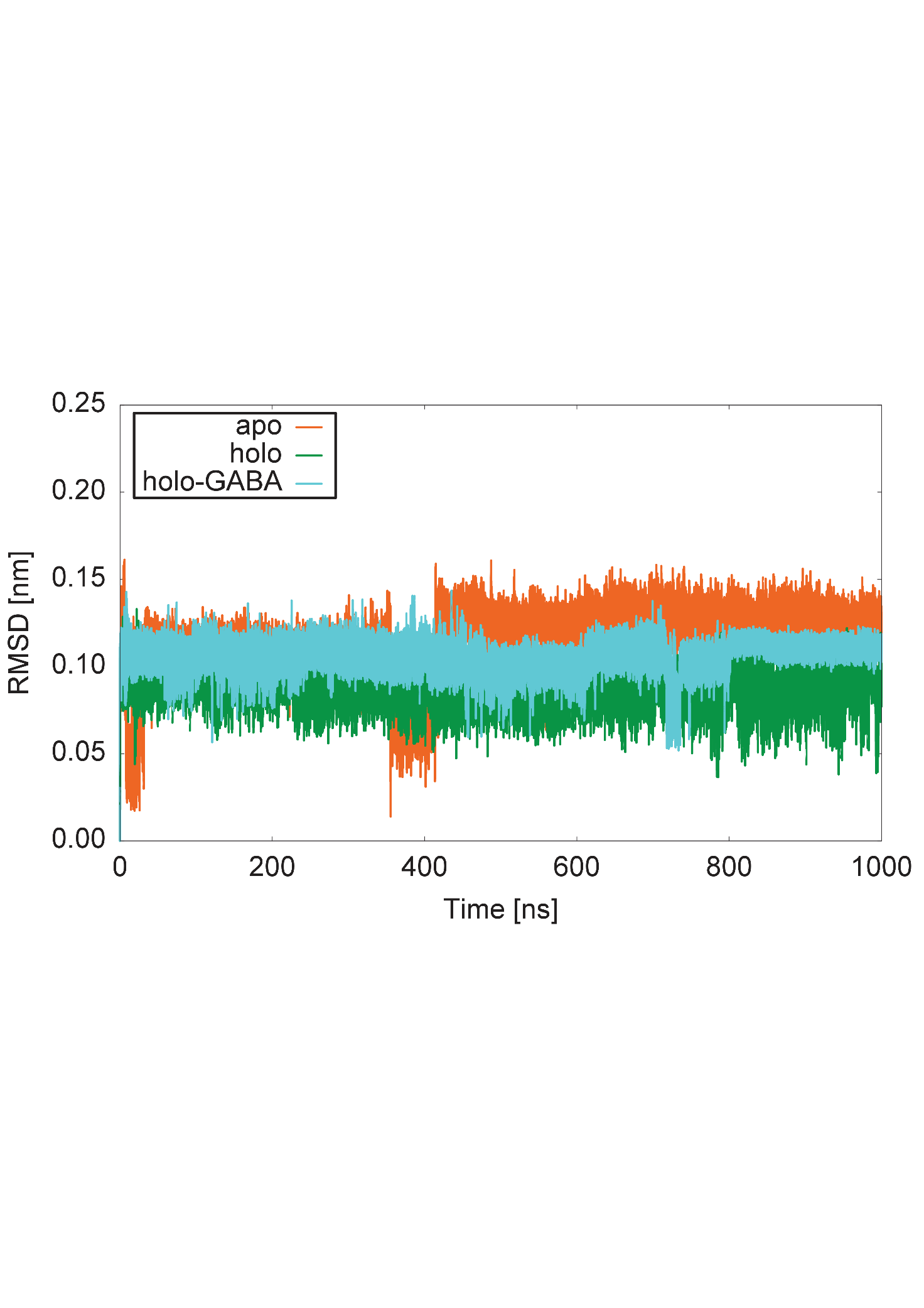
**

A

**
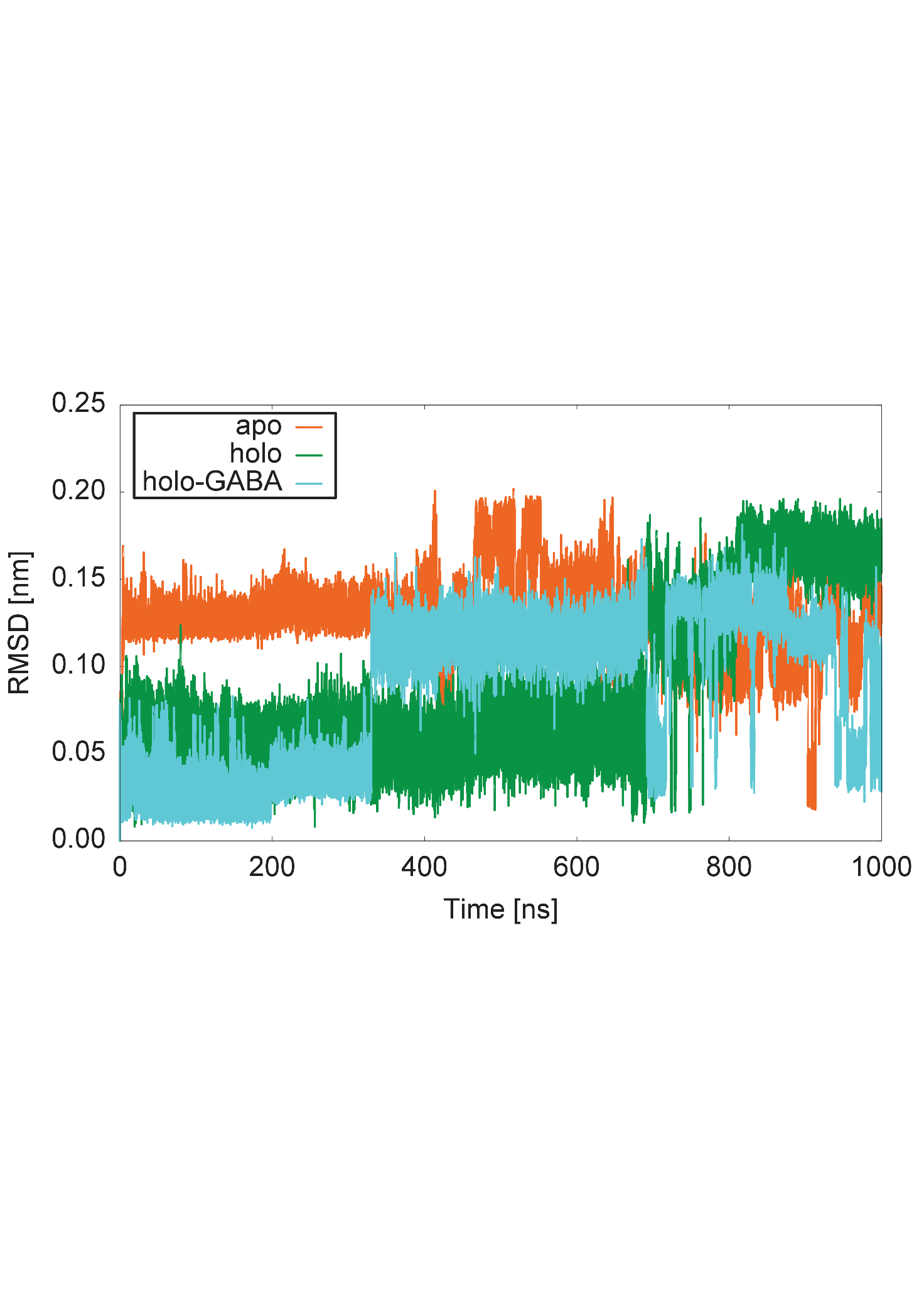
**

B

**Supplementary Figure S6. Arg319**

RMSD plot of the residue Arg319 in chains A and B (A and B panels, respectively). Plots have been created with the software gnuplot v. 4.4 (http://www.gnuplot.info/).

**
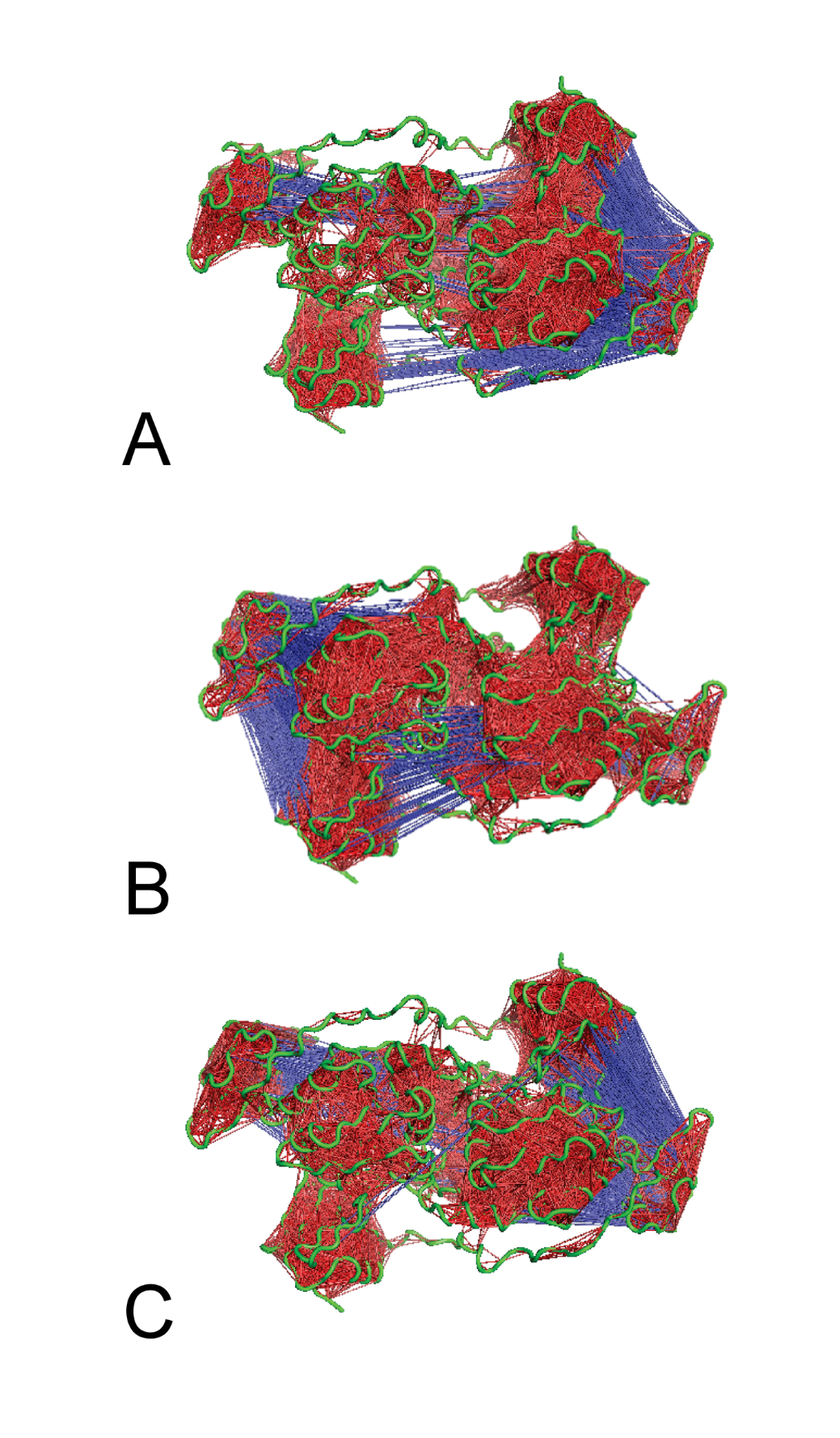
**

**Supplementary Fig. S7. Inter-residue correlations**

Inter-residue dynamics correlations mapped onto the apo (A), holo (B) and holo-GABA (C) GabR tridimensional structures. Only correlations with absolute value between 0.6 and 1 are shown. Positive and negative correlations are displayed as red and blue lines, respectively. The figure has been drawn with the program open-source PyMOL v. 1.8.4.0 (https://pymol.org/) using a Python script generated by the R package Bio3D (http://thegrantlab.org/bio3d/index.php).

**
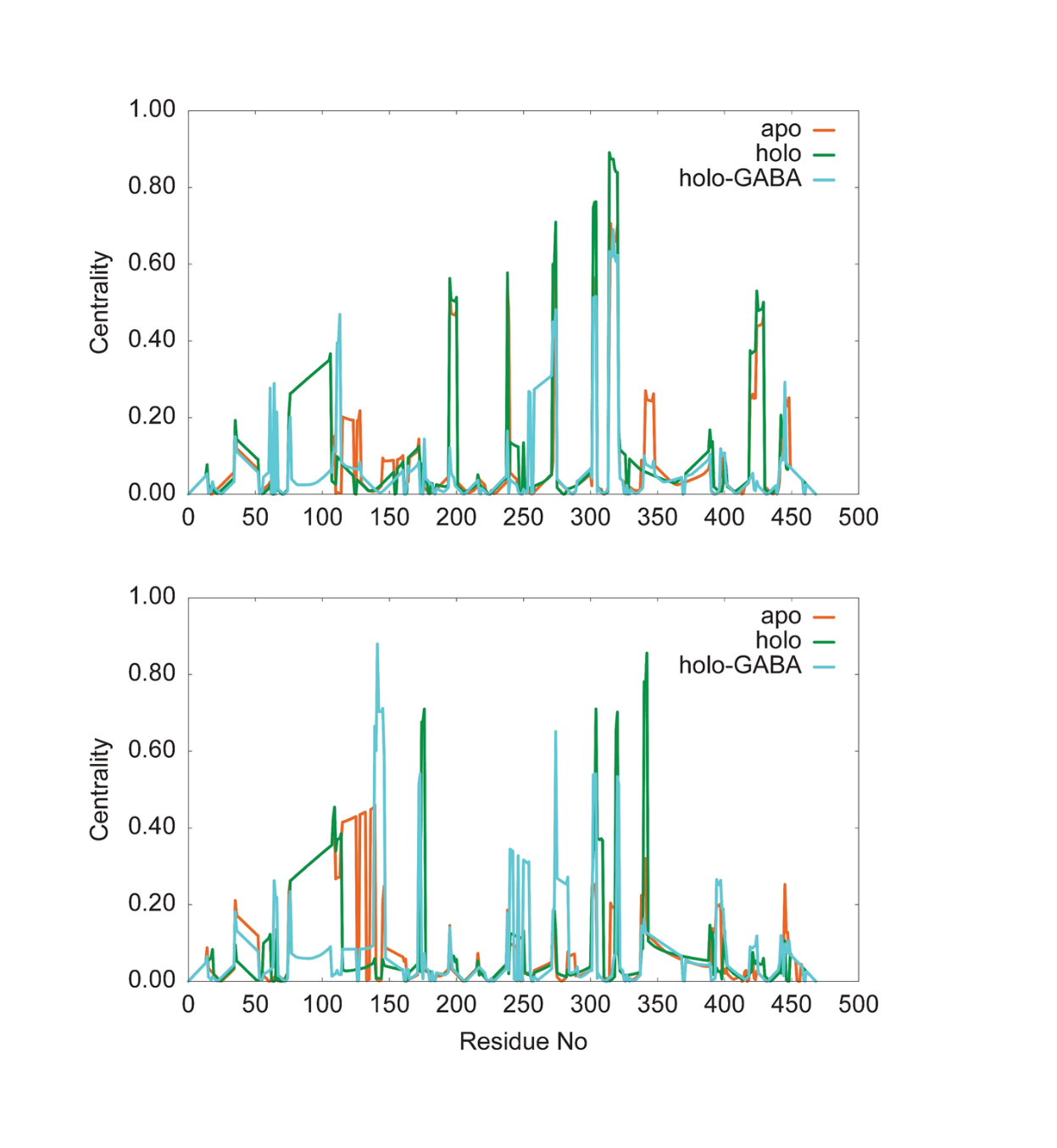
**

A

B

**Supplementary Figure S8. Betweenness**

Normalized betweenness (Centrality) of the three protein structure networks for (**A**) chain A and (**B**) chain B. Plots have been created with the software gnuplot v. 4.4 (http://www.gnuplot.info/).
